# Targeting mutant p53-R248W reactivates WT p53 function and alters the onco-metabolic profile

**DOI:** 10.3389/fonc.2022.1094210

**Published:** 2023-01-11

**Authors:** Kate Brown, Lisa M. Miller Jenkins, Daniel R. Crooks, Deborah R. Surman, Sharlyn J. Mazur, Yuan Xu, Bhargav S. Arimilli, Ye Yang, Andrew N. Lane, Teresa W-M. Fan, David S. Schrump, W. Marston Linehan, R. Taylor Ripley, Ettore Appella

**Affiliations:** ^1^ Laboratory of Cell Biology, Center for Cancer Research (CCR), National Cancer Institute (NCI), National Institutes of Health (NIH), Bethesda, MD, United States; ^2^ Urologic Oncology Branch, Center for Cancer Research (CCR), National Cancer Institute (NCI), National Institutes of Health (NIH), Bethesda, MD, United States; ^3^ Thoracic Surgery Branch, Center for Cancer Research, National Cancer Institute, National Institutes of Health, Bethesda, Maryland, United States; ^4^ Center for Environmental and Systems Biochemistry, Department of Toxicology and Cancer Biology, Markey Cancer Center, UK, Lexington, KY, United States

**Keywords:** mutant p53, metabolism, p53-R248W, gain-of-function, NSC59984

## Abstract

*TP53* is the most commonly mutated gene in cancer, and gain-of-function mutations have wide-ranging effects. Efforts to reactivate wild-type p53 function and inhibit mutant functions have been complicated by the variety of *TP53* mutations. Identified from a screen, the NSC59984 compound has been shown to restore activity to mutant p53 in colorectal cancer cells. Here, we investigated its effects on esophageal adenocarcinoma cells with specific p53 hot-spot mutations. NSC59984 treatment of cells reactivated p53 transcriptional regulation, inducing mitochondrial intrinsic apoptosis. Analysis of its effects on cellular metabolism demonstrated increased utilization of the pentose phosphate pathway and inhibition of glycolysis at the fructose-1,6-bisphosphate to fructose 6-phosphate junction. Furthermore, treatment of cells with NSC59984 increased reactive oxygen species production and decreased glutathione levels; these effects were enhanced by the addition of buthionine sulfoximine and inhibited by N-acetyl cysteine. We found that the effects of NSC59984 were substantially greater in cells harboring the p53 R248W mutation. Overall, these findings demonstrate p53-dependent effects of NSC59984 on cellular metabolism, with increased activity in cells harboring the p53 R248W mutation. This research highlights the importance of defining the mutational status of a particular cancer to create a patient-centric strategy for the treatment of p53-driven cancers.

## Introduction

Designated as a tumor suppressor, functions of the p53 protein expressed by the *TP53* gene include cell cycle arrest, apoptosis, and cellular senescence in response to DNA damage through both transcriptional and transcription-independent activities. *TP53* is the most commonly mutated gene in all cancers (1), and most mutations are gain-of-function (GOF) single missense mutations within the DNA-Binding Domain (DBD) (2) that can be categorized as affecting protein folding (conformational) or DNA binding (DNA-contact). Either of these may still allow for some wild-type (WT) p53 activity (3). The most frequent mutations are seen at six hot-spot codons: two DNA-contact residues, R248 and R273, and four conformational residues, R175, G245, R249, and R282 (4). The functional effects of *TP53* mutations include genome instability, increased invasion, proliferation, metastasis, and reduced apoptosis (5), as well as drug resistance, which could be responsible for reduced patient responses (6).

Adenocarcinoma of the esophagus (EAC) is the most common histological analysis for individuals afflicted with esophageal cancer in the United States (7). In contrast to most other cancers, including squamous cell carcinoma of the esophagus, EAC cases and death rates have been increasing over the past thirty years (8). The 5-year survival rate for EAC is under 20% irrespective of ethnicity, age, and disease stage at the time of diagnosis. *TP53* mutations occur in 50-70% of patients and are associated with disease progression and chemoresistance (9). Patients with EAC who harbor p53 mutations have a significantly worse outcome when treated with either neoadjuvant therapy or surgery alone (10), suggesting that mutant p53 may be a therapeutic target for EAC (9). The COSMIC database lists R248 as the most commonly mutated site for EAC samples, with 248Q and 248W mutations being observed. R175 is the second most common site of mutation in EAC (11). Previous studies have shown distinct differences between the p53 mutations R248W and R248Q in oral squamous cell carcinoma and non-small cell lung cancer (NSCLC), respectively, as well as differences between R175H and R175P (12, 13). Correlations between certain mutations of p53, disease progression and prognosis have also been made in breast cancer, colorectal cancer (CRC), and NSCLC (14–16).

In addition to its effects on cell growth, p53 has been shown to regulate multiple aspects of cellular metabolism. p53 transcriptionally controls the expression of SCO2 (17), a key regulator of the cytochrome c oxidase complex and oxidative phosphorylation (OXPHOS). Transcriptional regulation of GLS2, a mitochondrial glutaminase, drives glutaminolysis and subsequently OXPHOS by increasing glutamate and α-ketoglutarate levels (18). The regulation of glycolysis by p53 likewise has many components. The uptake of glucose into cells by GLUT1 and GLUT4 is directly regulated by p53 transcriptional control of these two proteins (19), whereas the regulation of GLUT3 is indirect *via* p53 modulation of NF-κB (20). TP53-induced glycolysis and apoptosis regulator (TIGAR) (21) is another transcriptional gene target of p53 that has a direct effect on glycolysis, converting fructose-2,6-bisphosphate to fructose-6-phosphate (22), decreasing glycolysis and diverting glucose into the pentose phosphate pathway (PPP). Here, p53 again plays a direct role in the pathway regulation by its interaction with glucose-6-phosphate dehydrogenase (G6PD), the rate-limiting enzyme of the PPP (23). The combination of these numerous effects also results in p53 regulating antioxidant levels to balance cellular redox (5). As p53 affects multiple aspects of cellular energy, p53 status may contribute to not only the response to standard therapeutics but also to differences in the metabolic profile and the cellular response to metabolic inhibition.

Inhibition of mutant p53 and reactivation of WT function is a strategy being pursued for development of new therapeutics. Although targeting p53 is not a new approach, previous trials have focused on identification of molecules that reactivate WT p53 function without also addressing the GOF effects seen in cancers. Since mutant p53 has multiple tumor-promoting effects, a successful treatment would ideally not only reactivate the normal function but also inhibit or degrade the remaining mutant protein. Several molecules have been reported to restore WT p53 function in tumors that express mutant p53 (24, 25). Of those, three have been shown to be effective against the R248 mutation. MIRA-1 prevents unfolding of both WT-p53 and mut-p53 and restores native WT-p53 conformation in R248Q (26). SCH529074 enables p53-R248W to bind to a consensus p53 DNA-binding site (27). Finally, RITA has been shown to restore transcriptional activity of both R248W and R248Q mutant p53 (28). Additional therapies have been shown to be successful in treating mut-p53 driven cancers without specifically targeting the mut-p53 itself. Examples include the statins that affect the mevalonate pathway (29); ganetespib, an HSP90 inhibitor (30); SAHA, an HDAC inhibitor (31); and COTI-2, a novel thiosemicarbazone derivative (32). The most clinically promising p53 reactivator to date is APR-246, an analog of PRIMA-1 in phase I and II clinical trials for a variety of cancer types including melanoma, ovarian, prostate and hematological cancers (33). APR-246 has been shown to inhibit tumor growth irrespective of p53 mutational status by overcoming chemoresistance and increasing cell cycle arrest and apoptosis (34–37). The APR-246 mechanism of action involves covalent modification of p53 cysteines 124 and 277 in the core domain, thereby increasing DNA binding, promoting correct folding, and subsequently recovering transcriptional activity (34, 36, 37). Additional p53-targeting molecules include the PK11000 family of molecules, arsenic trioxide (ATO) and NSC59984 (38–40). ATO was identified through a screen to identify cysteine-reactive compounds capable of rescuing structural p53 mutations (40). The family of PK11000 molecules was found to target cancer cells with mutant p53 by alkylating multiple cysteines, leading to increased regulation of p53 target genes (38). NSC59984 was first identified in a screen of the NCI Chemical Diversity Set II of compounds to find p53 reactivators. It was shown in colorectal cancer to restore WT p53 signaling and destabilize mutant p53 at non-genotoxic doses, with subsequent experiments in head and neck squamous cell carcinoma showing similar effects (39, 41).

Given the central effects of p53 on cellular metabolism, here we aim to investigate the effects of NSC59984 on mutant p53 and metabolism. We have analyzed the effects of NSC59984 inhibition of mutant p53 in patient-derived EAC cells and isogenic cell lines that express different forms of p53. Using both direct enzymatic assays and carbon tracing, we identified the p53-dependent pathways used for energy production and the functional effects that result from limiting these pathways though mutant p53 inhibition.

## Materials and methods

### Antibodies

Bax 1:1000 (AbCam), Bak1 1:1000 (AbCam), p21 1:1000 (Cell signaling), TP53 D01 1:200 (Santa Cruz), TP53 D01 ChIP Grade 1 μg/ul (AbCam), TP53 D07 1:100 (Millipore), TP73 1:200 (Santa Cruz), DAPI (10 nM) (Invitrogen), G6PD 1:1000 (AbCam), TIGAR 1:200 (AbCam), β-Actin 1:1000 (Santa Cruz), Anti-Mouse IgG HRP 1:5000 (Santa Cruz), Anti-Rabbit IgG HRP 1:5000 (Santa Cruz), Anti-mouse AF633 5 µg/ml (Invitrogen), Mouse IgG Isotype control 1:1000 (Santa Cruz), Rabbit IgG Isotype control 1:1000 (Santa Cruz), Mouse IgG2a ChIP Grade 1 μg/ul (AbCam).

### shRNA lentiviral transduction particles (Millipore Sigma)

Bax - SHCLNV-NM_001188 (TRCN0000312625)

Bak1 - SHCLNV-NM_004324 (TRCN0000033466)

Control - SHC001V

### CRISPR knockout gRNA lentivirus particles (Millipore Sigma)

TP53: HSPD0000042844 (LV01 – backbone)

Control: CRISPR12V CRISPR-Lenti Universal Non-Target Negative Control 1, Cas9+gRNA Lenti Particles (vector)

### P53 expression plasmids (Addgene)

WT: pCMV-Neo-Bam p53 wt was a gift from Bert Vogelstein (Addgene # 16434 https://n2t.net/addgene:16424; RRID : Addgene_16434)

R248W: pCMV-Neo-Bam p53 R248W was a gift from Bert Vogelstein (Addgene # 16437; https://n2t.net/addgene:16437; RRID : Addgene_16437)

### siRNA (Millipore Sigma)

TIGAR – SASI_Hs01_00070173

Control – MISSION universal Negative control #1

### RT-qPCR primers

β-Actin – Custom Probe and Primers (42)

Probe: 6FAM CCA GCC ATG TAC GTT GCT ATC CAG GC -TAMRA

Primer F - GCGAGAAGATGACCCAGATC

Primer R – CCAGTGGTACGGCCAGAGG

### Taqman primer probes (Invitrogen)

PUMA/BBC3: Hs00248075_m1

TP53: Hs01034249_m1

CaN19/S100A: Hs00195582_m1

PFKL: Hs01036347_m1

G6PD : Hs00166169_m1

### SYBR green primers

P21 F: GTGGCTCTGATTGGCTTTCTG

P21 R: CTGAAAACAGGCAGCCCAAG

TIGAR F: CCCCAGCAGATTGCAAAGA

TIGAR R: CAGCCGGCATCAAAAACAT

CaN19/S100A F: GGTCCAGGATGCCCAGTC

CaN19/S100A R: GAAGGAGAGCAAGGCAGC

### Cell culture

NCI-SB-EsC3 (EsC3) was established in the Thoracic Epigenetics Lab of Dr. David Schrump from a 59-year-old male non-smoker who developed malignant ascites approximately 1 year after ChemoRadioTherapy, esophagectomy, and adjuvant chemotherapy at an outside institution for stage III EAC. The cell line identity was confirmed using HLA typing and pathologic assessment relative to the patient’s primary tumor.

Barrett’s esophagus cell line CP-A was purchased from ATCC; the remaining cell lines were purchased from ECACC. Cells were maintained in either RPMI supplemented with 10% FBS (HyClone), 1% Penicillin–Streptomycin (Sigma) and 0.1% Amphotericin B (Gibco) or CP-A cells were maintained in the complete media recipe according to ATCC.

The sequence for human *TP53* was confirmed by Genewiz^®^ SNP Discovery/Mutation analysis of 22 human gDNA samples of Homo sapiens TP53 (NM_001126112.2) exonic regions 2 to 11, covered by up to 13 amplicons. Assays are designed to include at least 10 bp of flanking sequence at 5’ and 3’ ends relative to the exon sequences.

shRNA knockdown cells were transduced with shRNA to a final MOI of 5 in 12-well plates. Cells were incubated for 48 hr following transduction before addition of selection media. Cells were kept in selection media for one week before confirmation of gene expression by mRNA and western blot. Once sufficient knockdown of gene expression had been confirmed, further analysis was carried out.

siRNA knockdown cells were transiently transfected with 500 ng siRNA in 12-well plates in media containing no serum or antibiotics. Cells were incubated for 6 hr following transfection before addition of complete media. Cells were kept in media for 72 hr before confirmation of gene expression by mRNA and western blot. Once sufficient knockdown of gene expression had been confirmed, further analysis was carried out.

CRISPR knockout cells were transduced with shRNA to a final MOI of 5 in 12-well plates. Cells were incubated for 48 hr following transduction before addition of selection media. Cells were kept in selection media for one week before confirmation of gene expression by mRNA and western blot. Once sufficient knockdown of gene expression had been confirmed, further analysis was carried out.

Treatments with NSC59984 ((E)-1-(4-methylpiperazin-1-yl)-3-(5-nitrofuran-2-yl)prop-2-en-1-one) were carried out in complete RPMI media at 12 µM for 72 hr unless otherwise stated. DMSO was used as a carrier control for all treatments.

### Extracellular flux analysis

Cellular respiration was measured using the Seahorse XF96 Flux Analyzer and the XF Cell Mito Stress Test kit according to the manufacturer’s instructions (Agilent). Cells were seeded onto Poly-D-Lysine coated plates. The oxygen consumption rate (OCR) was measured under basal conditions and in response to the ATP synthetase inhibitor, Oligomycin (2.5 μM), the electron transport chain (ETC) accelerator, FCCP (1 μM), and finally the ETC complex 1 and 3 inhibitors, Antimycin A and Rotenone (2.5 μM each). Addition of these compounds (represented by arrows on the readout) allow for the following calculations. Basal Respiration was calculated from the OCR data at 26 minutes, after subtracting the non-mitochondrial respiration determined after addition of Antimycin A and rotenone. ATP production was calculated by the change in OCR before and after injection of oligomycin. Maximal respiratory capacity was calculated after injection of FCCP, as the maximum OCR reading minus the non-mitochondrial respiration rate. Spare capacity was calculated by subtracting the basal respiration from the maximal respiration rate.

Cellular glycolytic function was measured using the Seahorse XF96 Flux Analyzer and the XF Glucose Stress Test kit according to the manufacturer’s instructions (Agilent). Cells were seeded onto Poly-D-Lysine coated plates. The Extracellular Acidification Rate (ECAR) was measured under basal conditions and in response to glucose (10 mM), the ATP synthase inhibitor, Oligomycin (2.5 μM), and finally the glycolysis inhibitor, 2DG (100 mM). Addition of these compounds (represented by arrows on the readout) allowed the following parameters to be calculated: Basal ECAR was calculated from the ECAR data at 26 minutes, by subtracting the non-glycolytic acidification after adding 2DG. Glycolysis was estimated as the increase in basal ECAR after the addition of glucose. Glycolytic capacity was calculated as the maximum ECAR reading after the addition of oligomycin subtracting non-glycolytic acidification. Glycolytic reserve was calculated by subtracting glycolysis from the glycolytic capacity.

Data was normalized to cell number following completion of the assay and analyzed using the XF software (Seahorse Bioscience, Agilent Technologies). Assays were completed at least three times with four biological replicates of each experiment. A 2-way ANOVA with Šidák’s correction was carried out for statistical analysis.

### Cell proliferation

The CyQuant^®^ Cell Proliferation Assay Kit (Life Technologies) was used according to the manufacturer’s instructions. Cells were seeded onto Poly-D-Lysine coated plates and stimulated for 72 hr, after which the medium was removed from the cells and replaced with fresh medium without drug. On five consecutive days the medium was removed from the cells and the plate stored at -80°C at least overnight to lyse the cells. Once plates from all five days had been lysed CyQuant reagents were added for 15 mins before samples were analyzed on the Synergy HI microplate reader (BioTek). Results were calculated from the standard curve and are represented as fold change over control. A 2-way ANOVA test with Tukey correction was carried out for statistical analysis.

### Apoptosis assay

The ApoAlert™ Annexin V-FITC Apoptosis Kit (Takara) was used according to the manufacturer’s instructions. Cells were seeded and stimulated for 72 hr. Analysis was carried out on a FACS Calibur (BD Biosciences). Quadrants were defined by single stain controls and the percentages of apoptotic cells were calculated from both early and late apoptotic fractions. A 2-way ANOVA test with Tukey correction was carried out for statistical analysis.

### Western blot and co-immunoprecipitation

Cells were lysed with freshly prepared RIPA buffer containing 50 mM Tris–HCl, 10 mM MgCl_2_, 20% glycerol and 1% Triton X-100 with protease and phosphatase inhibitors (Roche) and centrifuged at 10,000 × *g* for 10 mins at 4°C. Protein concentration was determined by BCA assay (Pierce). Samples for Western blot analysis were combined with NuPAGE LDS sample buffer (Life Technologies) containing 0.05% β-mercaptoethanol, and incubated at 95°C for 5 minutes. 20 µg of protein was loaded onto a 4-12% SDS-Polyacrylamide gel. The resolved proteins were blotted onto nitrocellulose membrane and detected using the appropriate antibodies as shown in the reagents and β-actin served as the loading control for all western blots. Results were imaged using ECL and the Molecular Imager ChemiDoc Touch (BioRad).

Nuclear proteins were extracted using the NE-PER Nuclear and Cytoplasmic Extraction Reagents (Pierce) according to manufacturer’s instructions before BCA quantification.

4 mg of total protein or 500 µg nuclear protein were analyzed by immunoprecipitation. Samples were then pre-cleared with appropriate IgG before incubation with antibody-coated Dynabeads G (Novex). Following an overnight incubation, the unbound sample was removed, and the IP samples were separated from the Dynabeads using NuPAGE LDS sample buffer (Life Technologies) containing 0.05% β-mercaptoethanol, heated to 95°C. Samples were then resolved on a 4-12% SDS-Polyacrylamide gel and identified by western blot as described above.

### Reverse transcription and qPCR

RNA was extracted using the Qiagen RNeasy mini kit. 1 µg of cDNA was synthesized using iScript cDNA Synthesis Kit (BioRad) followed by quantification using TaqMan Fast Universal PCR Master Mix and the QuantStudio 6 Flex system (Applied Biosystems). C_t_ values were calculated from a standard curve of human cDNA and mRNA expression levels were normalized based on the expression of β-actin. Either a 2-way ANOVA test with Dunnett’s correction or A 2-way ANOVA test with Tukey correction was carried out for statistical analysis.

### Sequential ChIP

The Active Motif ChIP-IT Express^®^ protocol was followed for sequential ChIP analysis of p21 at the p53 promoter region, as measured by SYBR green qPCR. Following 2 hr treatment with either carrier control or NSC59984, cells were fixed and cross-linked in 1% formaldehyde, the DNA was sheared using sonication to generate DNA fragments with an average length of 200 to 800 bp. Fragments were purified, quantified and analyzed on an agarose gel for shearing efficiency. ChIP assays were set up with 20 μg of chromatin in triplicate and 4 μg of either p53 or IgGm. Following the first ChIP chromatin was desalted and a second sequential ChIP was set up with 4 μg of p53 or IgGm. Cross-links were then reversed, and the DNA was purified and analyzed by SYBR green qPCR. Chromatin input was diluted 1:100 before analysis (left panel), ChIP DNA was analyzed as % of input (right panel). A 2-way ANOVA with Šidák’s correction was carried out for statistical analysis.

### Immunofluorescence

Cells were grown and stimulated on 18-mm glass coverslips for 72 hr. Cells were then fixed using 5% paraformaldahyde, permeabilized using 0.5% Triton X-100, counter stained with Hoechst 33342 at 10 nM. TP53 antibody was diluted 1:50 before incubation on fixed cells, and a secondary anti-mouse AF633 antibody used at 5 µg/ml before counter staining. Proteostat^®^ Aggresome detection kit (Enzo) was used to counterstain protein aggregates.

Images were captured on a Zeiss LSM710 confocal microscope capturing images at optimum slice depth for each sample. Gain settings and exposure times were kept constant between samples.

### DCFDA (ROS production)

The Cellular Reactive Oxygen Species Detection Assay Kit (AbCam) was used according to the manufacturer’s instructions. Cells were seeded and stimulated for 72 hr before the assay was carried out with 25 μM DCFDA. Samples were analyzed on a Synergy HI microplate reader (BioTek). Results were first normalized to a positive and negative control.

### Glutathione

The Glutathione Assay Kit (Sigma) was used according to the manufacturer’s instructions. Cells were seeded and stimulated for 72 hr. Samples were analyzed alongside a standard curve on a Synergy HI microplate reader (BioTek). Results were calculated from the standard curve and results are represented as fold change over control.

### G6PD activity assay

A Glucose-6-Phosphate Dehydrogenase Activity Assay Kit (Cell Signaling) was used according to the manufacturer’s instructions. Cells were seeded and stimulated for 72 hr. Samples were analyzed on a Synergy HI microplate reader (BioTek). Results were first normalized to a positive and negative control.

### Total NADPH

A NADP^+^/NADPH Quantification Kit (BioVision) was used according to the manufacturer’s instructions. Cells were seeded and stimulated for 72 hr. Samples were filtered through a 10 kDa molecular weight cut-off filter before analysis. Samples were analyzed alongside a standard curve on the Synergy HI microplate reader (BioTek). Results were calculated from the standard curve and normalized to total protein concentration; results are represented as fold change over control.

### PFK activity

A Phosphofructokinase Activity Kit (Sigma) was used according to the manufacturer’s instructions. Cells were seeded and stimulated for 72 hr. Samples were analyzed on the Synergy HI microplate reader (BioTek). Results were first normalized to background readings and results were calculated from the standard curve; results are represented as fold change over control.

### Hexokinase activity

A Hexokinase Assay Kit (AbCam) was used according to the manufacturer’s instructions. Cells were seeded and stimulated for 72 hr. Samples were analyzed on the Synergy HI microplate reader (BioTek). Results were first normalized to background readings and results were calculated from the standard curve; results are represented as fold change over control.

### Membrane potential

Cells were grown and stimulated for 72 hr before incubation with JC1 (eBioscience) at 2 µM for 2 hr diluted in phenol-red free media. The stain was removed and replaced with 1 mL phenol red-free media and incubated at 37°C for 10 mins before resuspension in FACS buffer (PBS + 0.5% FCS) and analysis on the FACSCalibur flow cytometer (BD).

### Lactate production assay

A Lactate Assay Kit (BioVision) was used according to the manufacturer’s instructions. Cells were seeded and stimulated for 72 hr, after which the media was removed from the cells and stored at -80°C at least overnight to inhibit the lactate dehydrogenase. Samples were analyzed alongside a standard curve on the Synergy HI microplate reader (BioTek). Results were calculated from the standard curve and normalized to total protein concentration; results are represented as fold change over control.

### Glucose uptake

The Glucose Uptake-Glo Assay (Promega) was used according to the manufacturer’s instructions. Cells were seeded and stimulated for 24 hr. Samples were analyzed on the Synergy HI microplate reader (BioTek). Results were first normalized to a positive and negative control.

### YSI

Cells were seeded with corresponding wells containing media only in 24-well plates. They were stimulated for 24 hr before media was changed to complete media without drug. Following a further 48 hr incubation, 300 μL of media was removed and placed into 1.5 mL tubes. The YSI 2950D Biochemistry Analyzer (YSI #52760) was used to measure glucose, glutamine, lactate, and glutamate content. All values were normalized to DNA content and subsequently to media only conditions.

### Stable isotope resolved metabolomics

Tracer experiments were carried out in triplicate. Following a standard treatment protocol, cells were switched into RPMI containing 25 mM ^13^C_6_-glucose with 2 mM glutamine and 10% dialyzed FBS, without added sodium pyruvate for 24 h. At the end of the tracing period, cells were washed 3 times in cold PBS, quenched in cold acetonitrile, and extracted in acetonitrile:water:chloroform (2:1.5:1) (43). The upper polar metabolite fractions were frozen in several aliquots each and lyophilized overnight. One fraction of the resulting residue was reconstituted in 55 µL of D_2_O containing DSS-d6^1^ and transferred to disposable 1.7 mm glass NMR tubes (Wilmad) for analysis, as described below. IC-UHR-FT-FTMS analyses were performed as described below (44), to assess ^13^C incorporation into various metabolites. For metabolites quantified with purified standards, data are expressed as nmol/mg protein. For metabolites for which a purified standard was not available for quantification purposes, compounds were identified by their exact mass, and data are expressed in fold change of ion intensities normalized to the internal standard (DSS).

The polar extracts were reconstituted in nanopure water before analysis on a Dionex ICS-5000+ ion chromatography system interfaced with a Thermo Fusion Orbitrap Tribrid mass spectrometer (Thermo Fisher Scientific) as previously described (44) using a *m/z* scan range of 80-700. Peak areas were integrated and exported to Excel *via* the Thermo TraceFinder (version 3.3) software package before natural abundance correction (45). The isotopologue distributions of metabolites were calculated as mole fractions as previously described (46). The number of moles of each metabolite was determined by calibrating the natural abundance-corrected signal against that of authentic external standards. The amount was normalized to the amount of extracted protein and is reported in nmol/mg protein.

Polar extracts reconstituted in D_2_O (> 99.9%, Cambridge Isotope Laboratories, Tewksbury, MA) containing 0.5 mmol/L d_6_-2,2-dimethyl-2-silapentane-5-sulfonate (DSS) as internal standard were analyzed by 1D ^1^H and ^1^H-HSQC NMR on a 14.1 T DD2 NMR spectrometer (Agilent Technologies, Santa Clara, CA). 1D ^1^H spectra were acquired using the standard PRESAT pulse sequence with 512 transients, 16384 data points, 12 ppm spectral width, an acquisition time of 2 s and a 6 s recycle time with weak irradiation on the residual HOD signal during the relaxation delay. The raw fids were zero filled to 131072 points and apodized with 1 Hz exponential line broadening prior to fourier transformation. 1D HSQC spectra were recorded with an acquisition time of 0.25 s with GARP decoupling and recycle time of 2 s over a spectral width of 12 ppm, with, 1024 transients. The HSQC spectra were then apodized with unshifted Gaussian function and 4 Hz exponential line broadening and zero filled to 16384 data points before Fourier transformation. Metabolites were assigned by comparison with in-house (47) and public NMR databases. Metabolite and their ^13^C isotopomers were quantified using the MesReNova software (Mestrelab, Santiago de Compostela, Spain) by peak deconvolution. The peak intensities of metabolites obtained were converted into molarity by calibration against the peak intensity of DSS (27.5 nmol) at 0 ppm for ^1^H spectra and that of phosphocholine at 3.21 ppm (molar mass determined from 1D ^1^H spectra) for HSQC spectra before normalization with mg protein in each sample.

### Statistical analyses

The results shown were obtained from at least three independent experiments. Results were plotted and analyzed with GraphPad Prism. Error bars show SD. Significance tests are described in individual methods. p-Values smaller than 0.05 were indicated as significant.

## Results

### Reactivating p53 signaling

**Figure 1:**
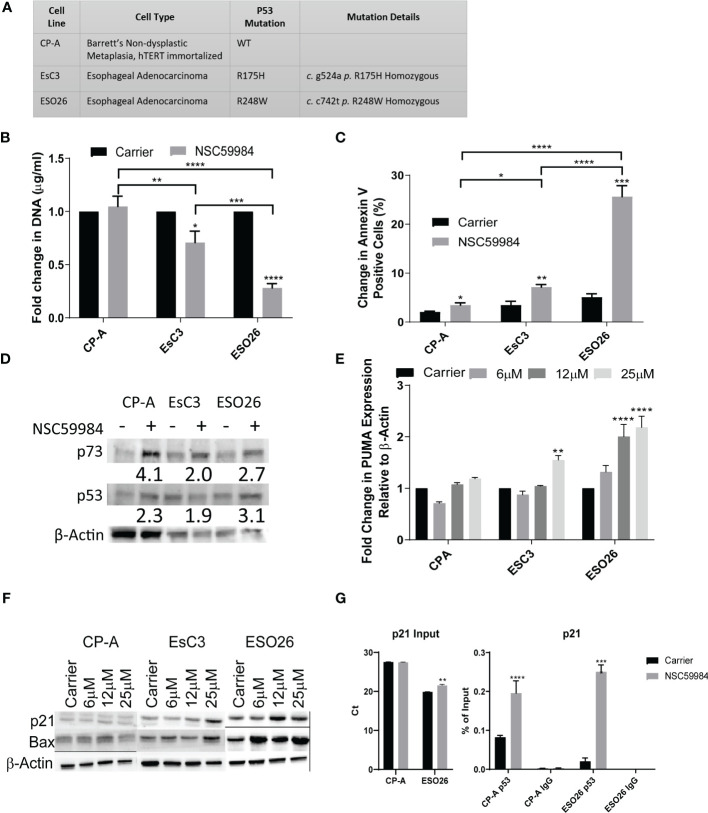
NSC59984 treatment re-activates WT-p53 signaling and intrinsic apoptosis. **(A)** Panel of EAC cells studied, providing their biological origin and associated *TP53* status. **(B)** Cellular proliferation was determined by fold change in CyQUANT measurement of cellular DNA following treatment with NSC59984 (12 μM) for 72 hr over carrier control in EAC cells. Fold change in carrier-treated cells was normalized to 1. A 2-way ANOVA test with Tukey correction was carried out for statistical analysis. **(C)** Analysis of apoptosis in EAC cells as measured by change in percentage of Annexin V positive cells following treatment with NSC59984 (12 μM) for 72 hr. A 2-way ANOVA test with Tukey correction was carried out for statistical analysis. **(D)** Whole cell lysate from EAC cells was blotted for p53 and p73 expression following 8 hr treatment with NSC59984 (12 μM). β-Actin served as the loading control. Numbers represent fold change in expression relative to changes in β-Actin. **(E)** Fold change in mRNA expression of PUMA analyzed by RT**-**qPCR following 3 hr treatment NSC59984 (6, 12 or 25 μM). Expression was normalized to β-actin, and changes in carrier-treated cells were set to 1. A 2-way ANOVA test with Dunnett’s correction was carried out for statistical analysis. **(F)** Whole cell lysate from EAC cells was blotted for Bax and p21 expression following 8 hr treatment with NSC59984 (6, 12 or 25 μM). β-Actin served as the loading control. **(G)** ChIP analysis of the occupancy of p53 on the p21 promoter in carrier-treated cells or following 2 hr treatment with 12 μM NSC59984 in CP-A-WT or ESO26-R248W cells. ChIP was performed using the Active Motif ChIP-IT Express^®^ kit. Chromatin Input was diluted 1:100 before analysis (left), ChIP DNA was analyzed as percent of Input (right). Raw Ct values can be found in Supplemental Table 1. A 2-way ANOVA with Šidák’s correction was carried out for statistical analysis. * = 0.05, ** = 0.005, *** = 0.0005, **** = 0.00005.

As p53 mutations are relatively common in EAC and are correlated with disease progression, we investigated their effects in patient-derived EAC cells. Sequencing all available patient-derived EAC cell lines revealed that four expressed hot-spot mutations (40%): EsC3, ESO26, ESO51 and OACM5.1 (data not shown). One high-grade, preneoplastic Barrett’s cell line (CP-C) was found to express R248W mutant p53, supporting the finding that p53 is an early stage mutation within EAC progression (48). Two other Barrett’s lines and normal esophageal cells expressed WT p53. A variety of p53 mutations were observed among the remaining cell lines, including both DNA binding and protein conformation mutations. Based on p53 status, three lines were selected for further study (**Figure 1A**, **S1A**): CP-A-WT; (low-grade Barrett’s cell line), EsC3-R175H (protein conformational mutation) and ESO26-R248W (DNA-binding mutation).

Previous experiments with NSC59984 demonstrated that it inhibits mutant p53 in CRC cells (39, 49). To determine whether these effects are also observed in EAC cells, we first investigated the effects of NSC59984 on p53 transcriptional activity. Treatment of mutant p53 cells with NSC59984 significantly reduced cellular proliferation (**Figure 1B**) and increased apoptosis as measured by Annexin V, as compared with treated CP-A-WT cells and each respective line, with ESO26-R248W cells showing 3-fold higher responses to treatment than EsC3-R175H cells (**Figure 1C**).

Mutant p53 protein is stabilized compared to the WT protein, which is rapidly degraded through the MDM2 pathway (50). Other p53 reactivators such as PRIMA-1 have been shown to alter p53 protein levels (51). We found that total protein levels of both p53 and, to a greater extent, p73 were increased in all EAC cell lines following treatment with NSC59984 (**Figure 1D**). Although the level of β-Actin differed between the three cell lines, within a line, it was unaffected by NSC59984 treatment. Whereas WT p53 is predominantly nuclear, mutant p53 aggregates in the cytoplasm, preventing it from entering the nucleus and promoting transcription (52). Immunofluorescence imaging was performed to determine if NSC59984 treatment affected p53 subcellular localization (**Figure S1C**). In untreated CP-A-WT cells, only nuclear p53 was observed, as shown by the overlap of nuclear stain DAPI (blue) with p53 stain (red); in EsC3-R175H and ESO26-R248W cells, p53 was detected in both the nucleus and in large clusters in the cytoplasm (red puncta marked with arrows in **Figure S1C**), suggesting it may aggregate there. Treatment with NSC59984 dissociated the cytoplasmic clusters in ESO26-R248W cells but had no effect on the more prevalent aggregates seen in EsC3-R175H cells (**Figure S1C**, right). To confirm that the cytoplasmic clusters of p53 were aggregates, we examined colocalization of p53 and Proteostat (green), a marker of protein aggregation. We found that indeed Proteostat did co-localize with the cytoplasmic p53 clusters (yellow puncta generated by the overlap of the p53 (red) and Proteostat (green) stains), confirming that the clusters represent aggregates (**Figure S1D**). Thus, NSC59984 disrupts mutant p53 protein aggregates in the ESO26-R248W cells but has a minimal effect on those in EsC3-R175H cells.

We next investigated the reactivation of p53 transcriptional activity. NSC59984 treatment increased the level of *PUMA* mRNA (**Figure 1E**) and Bax protein (**Figure 1F**), both p53 transcriptional targets, in a dose-dependent manner, with the greatest increases observed in ESO26-R248W cells and lesser increases in EsC3-R175H and CP-A-WT cells. During intrinsic apoptosis, Bax translocates to mitochondria where it forms either a homodimer or a heterodimer with Bak1, leading to cytochrome *c* release and activation of the caspase cascade (53). Simultaneous shRNA knockdown of *BAX* and *BAK1* in ESO26-R248W cells abrogated the pro-apoptotic effects of NSC59984 (**Figure S1B**), indicating that apoptosis occurred *via* the mitochondrial intrinsic pathway. As in previous work (39), NSC59984 treatment of mutant p53 cells increased protein levels of CDKN1A/p21, a p53 transcriptional target, in a dose-dependent manner with the most pronounced effects observed in ESO26-R248W cells (**Figure 1F**). Chromatin immunoprecipitation (ChIP) analysis demonstrated a significant increase in p53 occupancy of the p21 promoter following treatment of ESO26-R248W cells with NSC59984 (**Figure 1G**). These results are consistent with the reactivation of WT p53 signaling and transcription in the mutant p53 cells.

It was apparent from our results that the ESO26-R248W cells were substantially more sensitive to NSC59984 treatment compared to EsC3-R175H cells. As mutations at R248 seem to occur at a higher frequency in EAC (57 counts compared to 43 on the COSMIC database), we focused our research on this predominant population (11, 54).

### Analysis of the cellular metabolic profile of EAC cells

**Figure 2:**
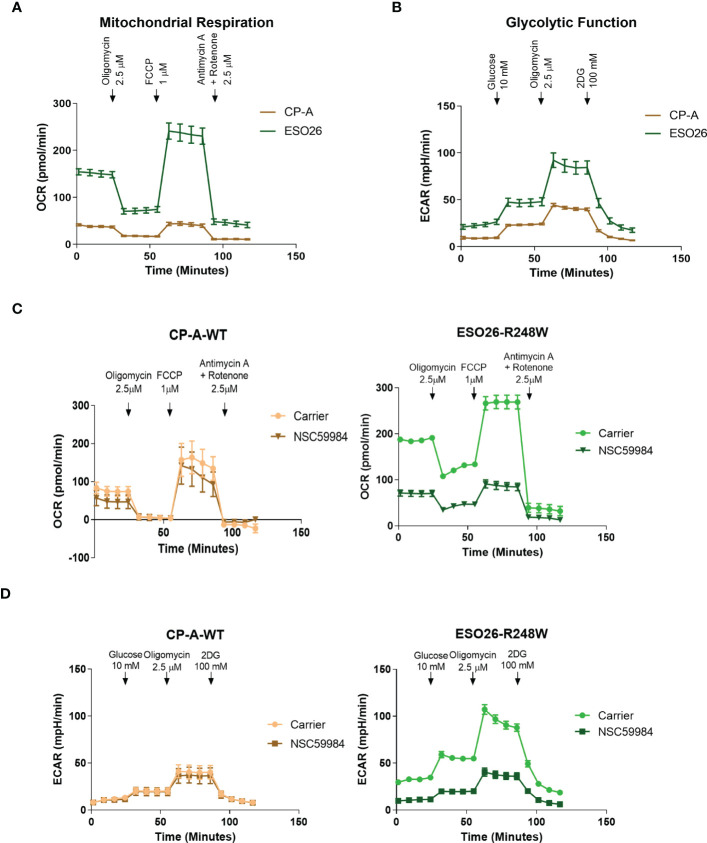
GOF mutant p53 alters cellular metabolic profiles. **(A)** Basal mitochondrial respiration, as measured with the Seahorse XF^e^96 Cell Mito Stress Test Kit, in the panel of EAC cells. Arrows indicate addition of metabolism-modulating drugs. **(B)** Basal glycolytic function, as measured with the Seahorse XF^e^96 Glycolytic Stress Test Kit, in the panel of EAC cells. Arrows indicate addition of metabolism-modulating drugs. **(C)** Mitochondrial function was measured using the Seahorse XF^e^96 Cell Mito Stress Test Kit following treatment with NSC59984 (12 μM) for 72 hr in EAC cells. **(D)** Glycolytic function was measured using the Seahorse XF^e^96 Glycolytic Stress Test Kit following treatment with NSC59984 (12 μM) for 72 hr in EAC cells. * = 0.05, ** = 0.005, *** = 0.0005, **** = 0.00005.

Given that p53 plays a significant role in metabolic pathways (5), we investigated the bioenergetic differences in these cells. Basal metabolic profiling was carried out using the Seahorse XF^e^96 extracellular flux bioanalyzer. Oxygen consumption rates (OCR) were used to study OXPHOS (**Figure 2A**), allowing the calculation of multiple aspects of mitochondrial respiration after normalization to cell number (**Figure S2A**). The CP-A-WT cells had low levels of basal respiration compared to mutant p53 cells, with OCR measurements four-fold less than that of ESO26-R248W cells. CP-A-WT cells had low levels of ATP production, maximal respiration and mitochondrial spare capacity as compared to mutant cells. A similar pattern was observed in glycolytic function as measured by extracellular acidification rates (ECAR) (**Figure 2B** and **S2B**). CP-A-WT cells had the lowest levels of basal ECAR, glycolysis, glycolytic capacity, and glycolytic reserve. Thus, there are clear differences in the metabolic profiles of p53 WT and mutant cells, with ESO26-R248W cells being the most glycolytically active, suggesting that p53 mutations may have effects on cellular energy usage.

We next performed Seahorse bioanalyzer analyses of metabolism in the EAC cells upon treatment with NSC59984. Both mitochondrial respiration and glycolytic function were reduced by NSC59984 treatment of ESO26-R248W cells, whereas minimal effects were observed in the CP-A-WT (**Figures 2C-D**, **S2C-D**). As shown above, ESO26-R248W cells had higher basal levels of cellular energy production (**Figure 2A**), and NSC59984 treatment reduced both OCR and ECAR in these cells to levels comparable to those of CP-A-WT cells (**Figure 2C-D**). When the parameters of mitochondrial respiration were calculated from the OCR profiles, we observed a general decrease in all aspects following NSC59984 treatment (**Figure S2C**); the only exception was that no changes in spare capacity were observed in CP-A-WT cells. All glycolytic parameters calculated from the ECAR profiles showed large, significant changes upon NSC59984 treatment in the ESO26-R248W cells (**Figure S2D**), whereas either no change or very small, but statistically significant, reductions in glycolytic capacity and glycolytic reserve were observed in the CP-A-WT cells.

### Development of an isogenic cell line

**Figure 3:**
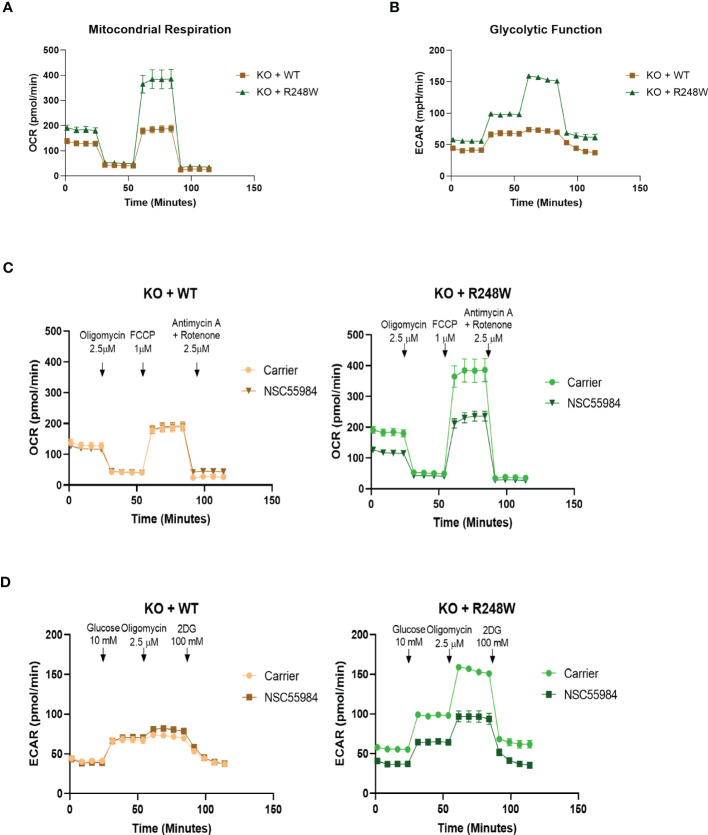
Isogenic cells match the parental cell lines. **(A)** Basal mitochondrial respiration, as measured with the Seahorse XF^e^96 Cell Mito Stress Test Kit, in p53-expressing KO cells. Arrows indicate addition of metabolism-modulating drugs. **(B)** Basal glycolytic function, as measured with the Seahorse XF^e^96 Glycolytic Stress Test Kit, in p53-expressing KO cells. Arrows indicate addition of metabolism-modulating drugs. **(C)** Mitochondrial function was measured using the Seahorse XF^e^96 Cell Mito Stress Test Kit following treatment with NSC59984 (12 μM) for 72 hr in p53-expressing KO cells. **(D)** Glycolytic function was measured using the Seahorse XF^e^96 Glycolytic Stress Test Kit following treatment with NSC59984 (12 μM) for 72 hr in p53-expressing KO cells. * = 0.05, ** = 0.005, *** = 0.0005, **** = 0.00005.

To determine whether the previously stated effects were due to the R248W mutation, we used CRISPR technology to knock-out (KO) p53 in the CP-A-WT cells and then stably reconstituted either p53-WT or p53-R248W (**Figures S3A-C**). It is interesting to note that although there are equal amounts of the p53 mRNA levels in these cells, the KO+R248W protein levels are substantially higher, indicating that the mutant protein is still stabilized compared to the WT protein, which is rapidly degraded through the MDM2 pathway. Using these stable isogenic cells, we first analyzed the effect of NSC59984 treatment on cellular proliferation (**Figure S3D**) and apoptosis (**Figure S3E**). We found that the pattern of response in the stable isogenic lines was the same as that observed in the EAC lines, with a significantly stronger response to NSC59984 observed in the KO+R248W cells. Next, we analyzed basal mitochondrial respiration (**Figure 3A**) and basal glycolytic function (**Figure 3B**). These data show metabolic profiles almost identical to the respective esophageal cell lines, with the KO+WT cells having much lower levels of both mitochondrial respiration and glycolytic function compared to the KO+R248W. These differences were confirmed following the calculation of the metabolic and glycolytic parameters where the KO+R248W cells showed higher levels in all measurements (Figure SF-G). Combined, these data show that the metabolic profiles of cells with exogenous mutant p53 have a metabolic pattern akin to that of the endogenous mutant p53 EAC cells, suggesting that the p53 mutation status of the cells plays an important role in their metabolic activity.

To test whether the effects of NSC59984 on cellular metabolism were due to the p53 mutation status, we also performed Seahorse bioanalyzer analyses of metabolism in the p53-expressing KO cells. The OCR and ECAR traces showed large decreases upon NSC59984 treatment in the KO+R248W cells, but minimal effects were observed in KO+p53WT (**Figures 3C, D**). These results are similar to the observed results in the EAC cells (**Figures 2C, D**). Calculation of the metabolic parameters show decreases in all aspects of mitochondrial respiration in the KO+R248W cells upon NSC59984 treatment, consistent with mutant p53 EAC cells (**Figure S3H**). Finally, as observed in the ESO26-R248W cells, all glycolytic functional parameters were decreased upon NSC59984 treatment in the KO+R248W cells (**Figure S3I**). Overall, these results indicate that, although not identical, the metabolic reaction to NSC59984 treatment in cells expressing p53-R248W endogenously or exogenously is very similar and suggests that this p53 mutation could play an important role in cellular metabolic functionality in tumors. It is important to note that in both the ESO26-R248W and KO+R248W cells, treatment with NSC59984 reduced the OCR and ECAR values to those of the CP-A-WT or KO+WT cells. This indicates that NSC59984-induced activation of WT-p53 activity also reverts the metabolism of p53 mutant cells to that of WT cells.

### Detailed analysis of cellular metabolism

**Figure 4:**
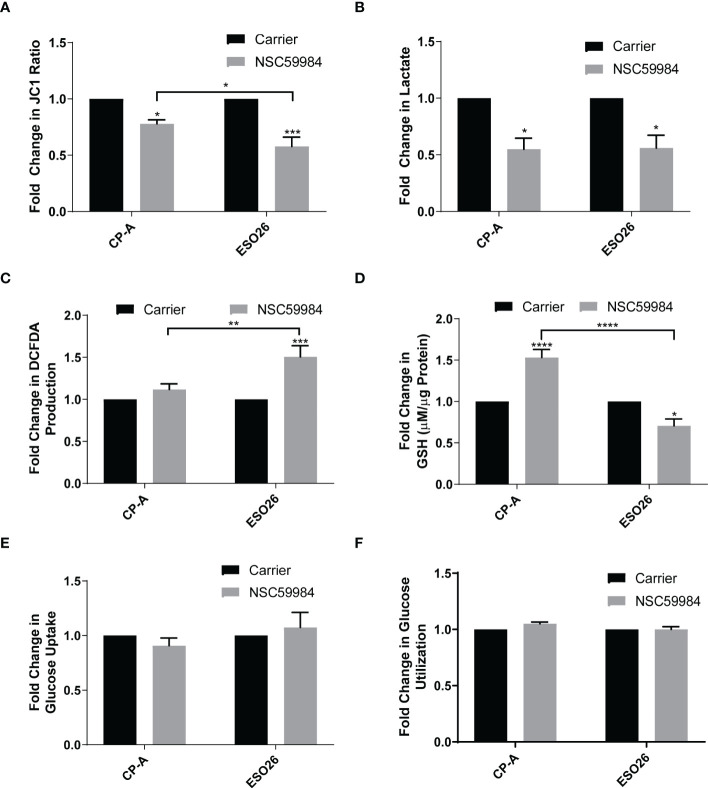
Effects of NSC59984 treatment on specific aspects of mitochondrial function. **(A)** Analysis of mitochondrial membrane potential (ΔΨm) as measured by the ratio of JC-1 fluorescence following treatment with NSC59984 (12 μM) for 72 hr over carrier control in EAC cells. Fold change in carrier-treated cells was normalized to 1. **(B)** Fold-change in lactate secretion (mM per µg of cellular protein) following treatment with NSC59984 (12 μM) for 72 hr over carrier control in EAC cells. Fold change in carrier-treated cells was normalized to 1. C. Fold-change in total ROS levels (DCFDA) following treatment with NSC59984 (12 μM) for 72 hr over carrier control in EAC cells. Fold change in carrier-treated cells was normalized to 1. **(D)** Fold-change in total GSH level (µM GSH per µg of cellular protein) following treatment with NSC59984 (12 μM) for 72 hr over carrier control in EAC cells. Fold change in carrier-treated cells was normalized to 1. **(E)** Fold-change in glucose uptake following treatment with NSC59984 (12 μM) for 72 hr in EAC cells. **(F)** Fold-change in glucose utilization following treatment with NSC59984 (12 μM) for 72 hr over carrier control in EAC cells. Fold change in carrier-treated cells was normalized to 1. YSI analyzer measurements were normalized to CyQUANT measurement of cellular DNA. **(A-F).** A 2-way ANOVA test with Tukey correction was carried out for statistical analysis for each of these assays. * = 0.05, ** = 0.005, *** = 0.0005, **** = 0.00005.

The changes to the metabolic profiles of EAC cells shown above indicated that further investigation into some of the more specific metabolic functions was needed. The reduced ATP production upon NSC59984 treatment indicates a lack of electron movement through the electron transport chain; this is supported by a reduction in mitochondrial membrane potential (ΔΨm), as measured by the ΔΨm-dependent JC-1 fluorescence indicator (**Figure 4A**), with a significantly stronger response seen in the ESO26-R248W cells than in the CP-A-WT. Both CP-A-WT and ESO26-R248W cells also showed similar significant reductions in lactate fermentation as measured by secreted lactate (**Figure 4B**). Likewise, KO+R248W cells had reduced lactate secretion following NSC59984 treatment; in contrast, KO+p53WT cells showed no response, unlike their parental cell counterparts (**Figure S4A**). ROS production, which is associated with impaired OXPHOS and glycolytic dysregulation (5), was increased by NSC59984 in ESO26-R248W and KO+R248W cells (**Figures 4C**, **S4B**), consistent with the high levels of apoptosis (**Figure 1C**, **S3E**) and oxidative stress (**Figures 2C**, **3C**) observed. In response to high levels of ROS, GSH is converted from oxidized glutathione to protect the cell from toxic redox activity. Consistent with the small, but not statistically significant increase in ROS in CP-A-WT cells upon NSC59984 treatment, GSH levels also increased (**Figure 4D**). In contrast, though there was significantly higher ROS in the ESO26-R248W cells after treatment with NSC59984, GSH levels actually decreased, suggesting dysregulation in the redox system in the ESO26-R248W cells. Similar results were seen in the KO cells, with those expressing WT p53 showing an increase in GSH and those expressing p53 R248W a significant reduction (**Figure S4C**). Reductions in both OXPHOS and glycolysis indicate a reduction in glucose availability. The glucose import channels GLUT1 and GLUT4 are downregulated by WT p53 and conversely upregulated by mutant p53 (5). We found that the total amount of glucose in the cell and its utilization over 24 hours following treatment were unaffected by NSC59984 (**Figures 4E, F**), indicating that glucose entered the cell but was not processed *via* glycolysis.

**Figure 5:**
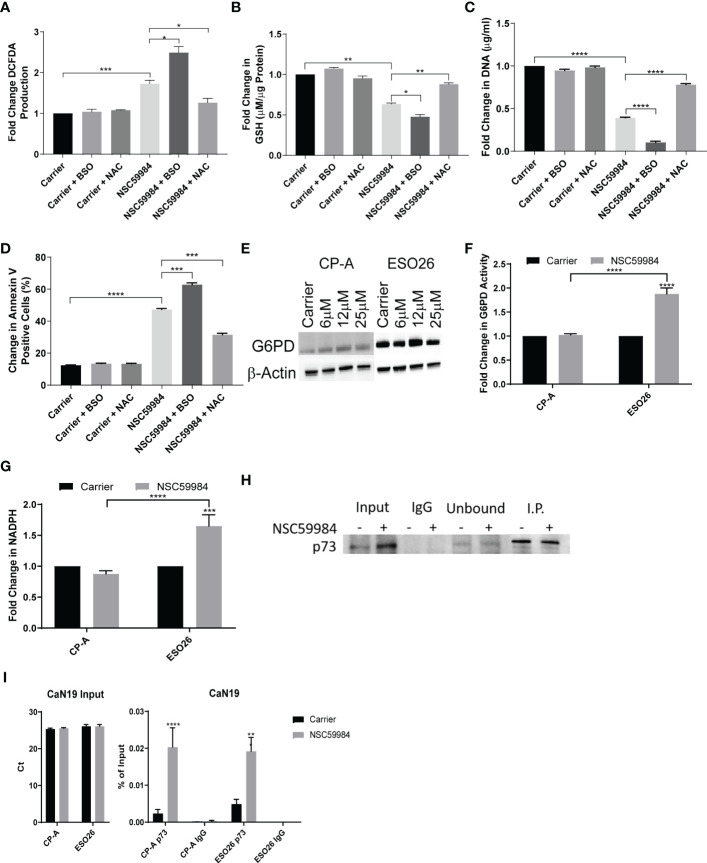
Modulation of NSC59984 effects by combination treatment with ROS regulators. **(A)** Fold change in total ROS levels (DCFDA) following treatment of ESO26-R248W cells with NSC59984 (12 μM) for 24 hr either alone or in combination with NAC (1 mM) or BSO (10 mM) over carrier control. Fold change in carrier-treated cells was normalized to 1 **(B)** Fold change in total GSH level (µM GSH per µg of cellular protein) in ESO26-R248W cells treated with NSC59984 (12 μM) for 24 hr either alone or in combination with NAC (1 mM) or BSO (10 mM) over carrier control. Fold change in carrier-treated cells was normalized to 1. **(C)**. ESO26-R248W cellular proliferation was measured following treatment with NSC59984 (12 μM) for 24 hr either alone or in combination with NAC (1 mM) or BSO (10 mM). Cells were re-seeded and fold change in CyQUANT quantitation of cellular DNA was measured following 5 days of growth over carrier control. Fold change in carrier-treated cells was normalized to 1. **(D)** Apoptosis in ESO26-R248W cells treated with NSC59984 (12 μM) for 24 hr either alone or in combination with NAC (1 mM) or BSO (10 mM) as measured by change in percentage of Annexin V positive cells. Cells were re-seeded and the change in percent of Annexin V positive cells was assessed following 72 hr growth. **(E)** Effect of NSC59984 treatment on G6PD protein level. Shown is a representative image, whole cell lysate was blotted for G6PD expression following 8 hr treatment with NSC59984 (12 μM) in EAC cells. **(F)** Fold change in G6PD activity following treatment with NSC59984 (12 μM) for 72 hr over carrier control in EAC cells. Fold change in carrier-treated cells was normalized to 1. **(G)** Fold-change in total NADPH levels following treatment with NSC59984 (12 μM) for 72 hr over carrier control in EAC cells. **(H)** Western blot analysis of p53-p73 protein levels. ESO26 cells were treated with NSC59984 (12 μM) for 72 hr before cell lysis. Total protein was IP with p53 D01. Sample Input, IgG preclear, unbound protein controls and IP samples were blotted for p73. Fold change in carrier-treated cells was normalized to 1. **(A-H).** A 2-way ANOVA test with Tukey correction was carried out for statistical analysis for each of these assays. **(I)** ChIP analysis of the occupancy of p73 on the CaN19 promoter in carrier-treated cells or following 2 hr treatment with 12 μM NSC59984 in CP-A-WT or ESO26-R248W cells. ChIP was performed using the Active Motif ChIP-IT Express^®^ kit. Chromatin Input was diluted 1:100 before analysis (left), ChIP DNA was analyzed as percent of Input (right). A 2-way ANOVA with Šidák’s correction was carried out for statistical analysis. Raw Ct values can be found in **Supplemental Table 2**. * = 0.05, ** = 0.005, *** = 0.0005, **** = 0.00005.

As APR-246 requires an oxidized environment to function (55), we decided to investigate whether the reduced cellular energetics and ROS produced in ESO26-R248W cells by NSC59984 treatment were also dependent on environmental conditions. Treatment with L-buthionine sulphoximine (BSO), an irreversible inhibitor of γ-glutamylcysteine synthetase, enhanced the increased ROS production in NSC59984-treated ESO26-R248W cells. Conversely, the ROS scavenger N-acetyl cysteine (NAC) inhibited the activity of NSC59984 (**Figure 5A**). A further decrease of GSH was observed when ESO26-R248W cells were treated with BSO in combination with NSC59984, whereas addition of NAC with NSC59984 increased GSH levels relative to treatment with NSC59984 alone to a level comparable with NAC treatment alone (**Figure 5B**). To determine whether the cellular redox state affected the downstream functional activity of NSC59984, proliferation and apoptosis were assayed in ESO26-R248W cells upon NSC59984 co-treatment with NAC or BSO. Consistent with our previous results, BSO enhanced the anti-proliferative and pro-apoptotic effects of NSC59984, which were inhibited by NAC (**Figures 5C, D**). Importantly, in all assays, neither BSO nor NAC had an effect when used alone. Thus, NSC59984 reduced global metabolism in ESO26-R248W cells, and its efficacy was enhanced when GSH levels were reduced in an oxidized environment.

As we observed a reduction in glycolysis, we investigated the activity of the PPP, an alternative to glycolysis for glucose metabolism. The first step of the oxidative branch Is conversion of glucose-6-phosphate (G6P) to 6-phosphogluconate by G6PD (56). We observed that G6PD mRNA and protein increased in response to NSC59984 treatment in ESO26-R248W cells but was unchanged in CP-A-WT cells (**Figures 5E**, **S5A**). In addition, G6PD activity was significantly increased by NSC59984 in ESO26-R248W cells (**Figure 5F**). A similar increase was seen in the KO+R248W cells, whereas only a minimal change was seen in the KO+p53WT cells (**Figure S5B**). In ESO26-R248W cells, a concurrent increase was observed in total cellular NADPH, produced by the activity of G6PD and 6PGDH, the third enzyme of the PPP (**Figure 5G**) (56). As WT p53 interacts directly with G6PD to inhibit its activity (23), we investigated changes in the protein-protein interaction upon NSC59984 treatment. Despite the changes in both protein level and activity, there was no change in the binding of G6PD to mutant p53 in NSC59984-treated ESO26-R248W cells and a only a small increase in CP-A-WT cells (**Figure S5C**).

G6PD is also transcriptionally regulated by the p53 family member p73 (57). Previous research on NSC59984, as well as studies on the mechanism of PRIMA-1, have demonstrated that reactivation of mutant p53 can result in destabilization of an inhibitory mutant p53-p73 complex, allowing p73 to be fully functional (39, 58). To determine whether this was also occurring in the ESO26-R248W cells, we pulled down p53 to examine p73 co-immunoprecipitation. Whereas the p73 protein level increased upon NSC59984 treatment (**Figure 1D**, **5H** input lanes), the level of p73 complexed with p53-R248W did not change (**Figure 5H**, I.P. lanes), suggesting that there is increased free p73 that would be transcriptionally available. In order to assess changes in p73 activity upon NSC59984 treatment, we looked at the mRNA expression of two genes transcriptionally regulated by p73 but not p53, CaN19 (59) and PFKL (60). Both were found to be increased in ESO26-R248W cells following treatment with NSC59984 (**Figures S5D-E**), indicating NSC59984 increases p73-dependent transcription. In addition, ChIP analysis demonstrated a significant increase in p73 occupancy of the CaN19 promoter following treatment of ESO26-R248W cells with NSC59984 (**Figure 5I**), indicating that the p73 that is not in a complex with p53 is transcriptionally active.

### Activating the PPP *via* TIGAR

**Figure 6:**
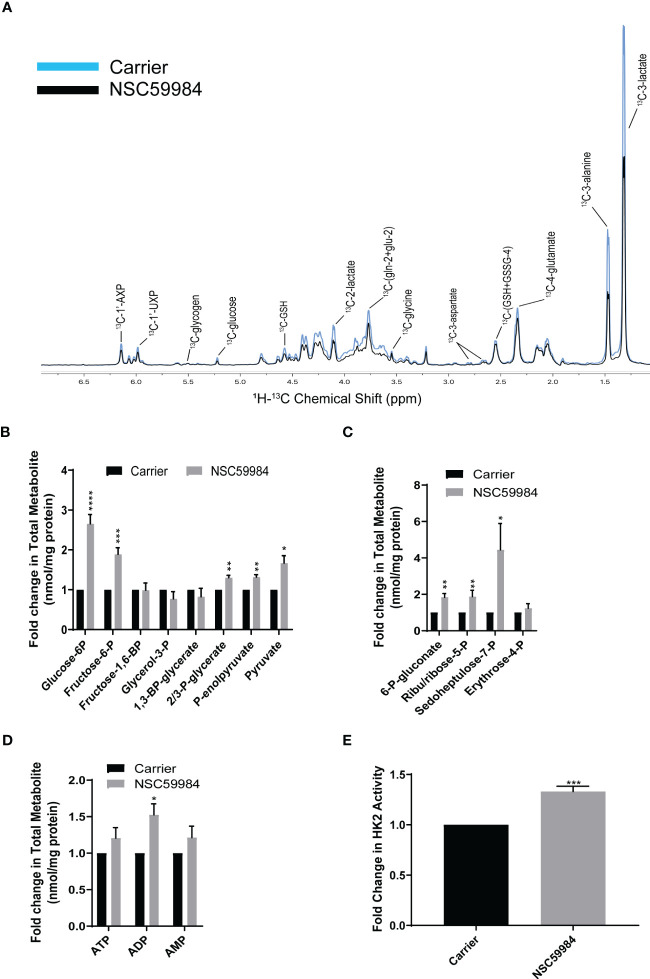
NSC59984 treatment results in activation of the PPP. **(A)** Incorporation of [^13^C_6_] glucose into cellular metabolites of ESO26-R248W cells following treatment with NSC59984 (12 μM) for 72 hr was assessed by ^1^H-^13^C HSQC NMR. Spectra were normalized to total cellular protein content. **B-D:** Fold change in total cellular quantities of metabolite intermediates were assessed by IC-FTMS over carrier control set to 1 in: **(B)** Glycolysis pathway; **(C)** Pentose phosphate pathway; **(D)** Adenylates ATP, AMP, and ADP. **(E)** Fold change in Hexokinase activity following treatment with NSC59984 (12 μM) for 72 hr over carrier control in ESO26-R248W cells. Fold change in carrier-treated cells was normalized to 1. **A-E.** Paired t-tests were carried out for statistical analysis for each of these assays. * = 0.05, ** = 0.005, *** = 0.0005, **** = 0.00005.

To further investigate PPP activation and how glucose was being utilized, we performed a tracer experiment with ^13^C_6_-glucose. NMR analysis of the polar extracts revealed few differences in the ESO26-R248W cells upon NSC59984 treatment (**Figure 6A**), confirming the lack of change in total cellular glucose metabolism. The two main differences observed were decreased lactate and alanine, consistent with the observed reductions in lactate excretion (**Figure 4B**) and glycolysis (**Figures 2D**, **S2D**). To further evaluate the processing of carbon though cellular metabolism, we performed IC-FTMS analysis of the same polar extracts. A nearly 3-fold increase in G6P and a subsequent 2-fold increase in fructose-6-P (F6P) were observed in ESO26-R248W cells (**Figure 6B**). When examining changes in metabolites of the PPP, ~2-fold increases in 6-P-gluconate and ribulose/ribose-5-P and a 4-fold increase in sedoheptulose-7-P were observed in ESO26-R248W cells (**Figure 6C**). Consistent with changes in the early glycolytic pathway upon NSC59984 treatment in ESO26-R248W cells, an increase in ADP, the product of glucose conversion to G6P, and a concurrent increase in the activity of HK2, the enzyme that drives this conversion, were observed (**Figures 6D, E**). There were no significant changes in labeling of Krebs cycle intermediates in the cells (**Figure S6A**). Consistent with the NMR analysis and direct measurements, lactate was reduced in ESO26-R248W cells without a significant change in the hexoses preceding it (**Figure S6B**). No significant changes were observed in GlcNAc-phosphate, UDP-GlcNAc or UDP-GalNAc, showing that glucose-derived carbon was not diverted (**Figure S6C**).

**Figure 7:**
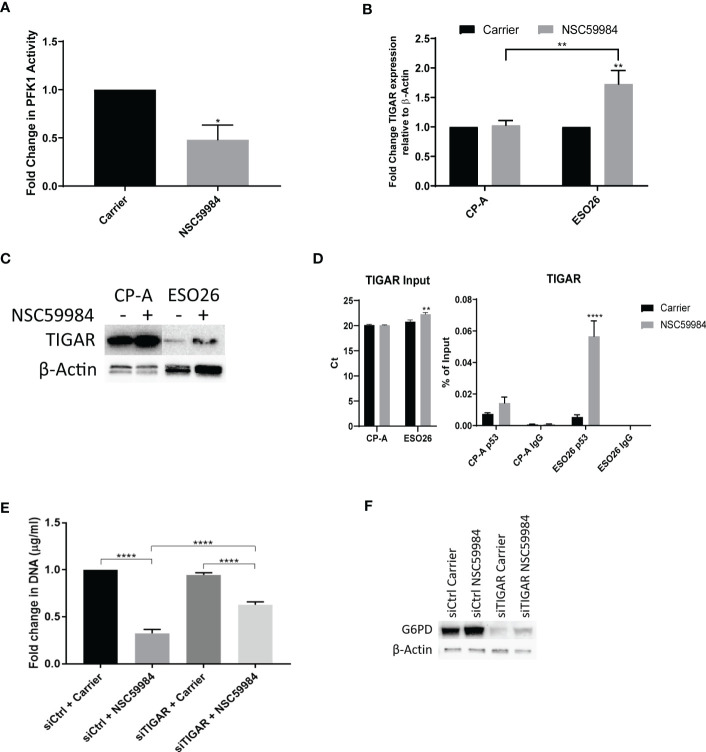
NSC59984 treatment results in p53-dependent activation of TIGAR. **(A)** Fold change in PFK1 activity following treatment with NSC59984 (12 μM) for 72 hr over carrier control in ESO26-R248W cells. Fold change in carrier-treated cells was normalized to 1. A paired t-test was carried out for statistical analysis. **(B)** Fold changes in mRNA expression levels of TIGAR were analyzed by RT**-**qPCR following treatment with NSC59984 (12 μM) for 72 hr over carrier control in EAC cells. Fold change in carrier-treated cells was normalized to 1. Expression was normalized to β-actin. A 2-way ANOVA test with Tukey correction was carried out for statistical analysis. **(C)** Whole cell lysate was blotted for TIGAR expression following treatment with NSC59984 (12 μM) for 72 hr in the selected panel of EAC cells. **(D)** ChIP analysis of the occupancy of p53 on the TIGAR promoter in carrier-treated cells or following 2 hr treatment with 12 μM NSC59984 in CP-A-WT or ESO26-R248W cells. ChIP was performed using the Active Motif ChIP-IT Express^®^ kit. Chromatin Input was diluted 1:100 before analysis (left), ChIP DNA was analyzed as percent of Input (right). A 2-way ANOVA with Šidák’s correction was carried out for statistical analysis. Raw Ct values can be found in **Supplemental Table 3** **(E)** Cellular proliferation in ESO26-R248W cells following treatment with NSC59984 (12 μM) for 72 hr over carrier control in siTIGAR or siControl cells. Proliferation was determined by change in CyQUANT measurement of cellular DNA. Fold change in carrier-treated cells was normalized to 1. A 2-way ANOVA test with Tukey correction was carried out for statistical analysis. **(F)** Western blot analysis of G6PD protein levels following treatment with NSC59984 (12 μM) for 72 hr in siTIGAR or siControl ESO26-R248W cells. * = 0.05, ** = 0.005, *** = 0.0005, **** = 0.00005.

A halt in glycolysis between F6P and fructose-1,6-bisphosphate (FBP) indicates reduced activity of phosphofructokinase-1 (PFK1). As PFK1 is inhibited by TIGAR, a gene that is transcriptionally regulated by p53 (5, 61), we examined whether there were any effects of NSC59984 treatment on PFK1 activity and TIGAR levels. Treatment of ESO26-R248W cells with NSC59984 led to a 2-fold decrease in PFK1 activity and increased *TIGAR* expression at both the mRNA and protein levels (**Figures 7A-C**). Given the p53 transcriptional control of TIGAR expression, it is interesting to note the difference in basal TIGAR protein levels between CPA-WT and ESO26-R248W cells which are consistent with reduced p53 transcriptional activity in the mutant cells. ChIP analysis showed that p53 occupancy of the *TIGAR* promoter increased upon treatment with NSC59984 in ESO26-R248W cells (**Figure 7D**), consistent with the observed increase in p53 transcription. *TIGAR* knockdown by siRNA (**Figure S7**) partially abrogated the anti-proliferative effects of NSC59984 in ESO26-R248W cells (**Figure 7E**) and reduced the expression level of G6PD (**Figure 7F**). Combined, these data indicate that NSC59984 treatment shunts glucose utilization away from the glycolytic pathway into the PPP in cells with p53-R248W due at least in part to increased TIGAR resulting from increased p53 transcription.

### Combinatorial effects of metformin and/or 2-deoxy-D-glucose with NSC59984

**Figure 8:**
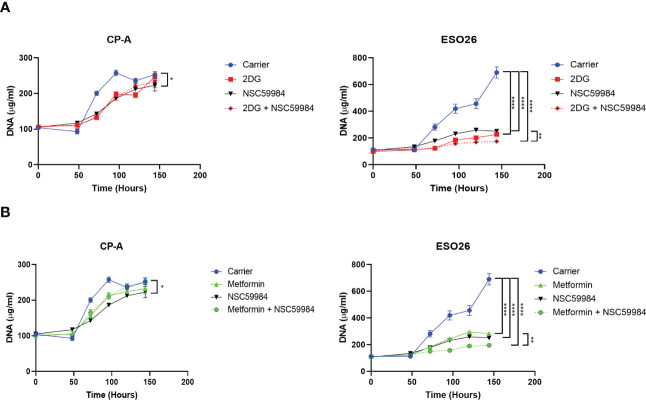
Combination of NSC59984 with metabolic inhibitors shows improved effects. **(A)** Analysis of cellular proliferation over a period of 5 days following 48 hr treatment with either Metformin (5 mM), NSC59984 (12 μM) or a combination in EAC cells **(B)** Analysis of cellular proliferation over a period of 5 days following 48 hr treatment with either 2DG (5mM), NSC59984 (12 μM) or a combination in EAC cells. In all panels, cellular proliferation was measured by CyQUANT analysis of cellular DNA. A 2-way ANOVA test with Tukey correction was carried out for statistical analysis. * = 0.05, ** = 0.005, *** = 0.0005, **** = 0.00005.

As NSC59984 treatment resulted in changes in cellular metabolism in the p53-R248W cells, we investigated whether combination with another metabolic inhibitor would further enhance its antiproliferative effects. We examined the effect of combined treatment of NSC59984 with metformin, an inhibitor of the OXPHOS pathway, or 2-deoxy-D-glucose (2DG), an inhibitor of the glycolytic pathway (62, 63). As shown previously for NSC59984 treatment of CP-A-WT cells, treatment with metformin did not significantly affect cellular proliferation, nor did combined treatment with metformin and NSC59984 (**Figure 8A**). In contrast, treatment with either metformin or NSC59984 alone reduced proliferation 75% in ESO-R248W cells, with combination treatment inhibiting it completely. Similar results were observed for the combination of NSC59984 with 2DG (**Figure 8B**). These results show that both metabolic inhibitors enhance the response of mutant p53 cells to NSC59984 but have little effect in the CP-A-WT cell line. Thus, combination of p53 reactivation with inhibition of either the OXPHOS or glycolytic pathways preferentially stops the growth of mutant p53 cells, suggesting a mechanism for therapeutic development.

## Discussion

Inactivating mutations of p53 are widespread across tumor types, making inhibition of mutant p53 gain-of-function with concurrent reactivation of wild type p53 activity a therapeutic goal. NSC59984 was initially reported to activate the p53 signaling pathway in CRC cells, and treatment resulted in increased expression of p53 transcriptionally-regulated genes and increased apoptosis without genotoxicity (39). Here, we have extended these findings in EAC cells, demonstrating for the first time the effects of NSC59984 on cellular metabolism. We observed that cellular metabolism was altered in EAC cells harboring mutant p53, as well as following NSC59984 treatment. Similar results were observed in isogenic cells exogenously expressing either WT or mutant p53-R248W. Of the p53 R175H and R248W mutants studied, larger effects of NSC59984 treatment were observed in cells with p53 R248W. Using CRISPR to generate isogenic cells allowed us to determine that the effects of NSC59984 were due to the p53 mutation specifically as opposed to other genetic differences between the lines. ESO26-R248W cells showed an overall reduction in global cellular energetics, and through enzymatic assays, direct measurement, and carbon tracing, we identified effects on the PPP. We additionally observed significant increases in NADPH (**Figure 5G**), a product of the oxidative branch of the PPP that is required for synthesis of fatty acids, deoxynucleotides needed for proliferation, as well as for removal of ROS (5). Active PPP likely decreases lactate production (23), consistent with the decreased lactate observed upon NSC59984 treatment. Increases in ADP level and TIGAR expression in the ESO26-R248W cells upon NSC59984 treatment also suggest increases in PPP activity. TIGAR functions like F2,6-bisphosphatase to reduce fructose-2,6-bisphosphate, the PFK1 activator, resulting in an accumulation of F6P, which subsequently isomerizes to G6P and enters the PPP (61). As TIGAR is transcriptionally regulated by p53, our overall observations support increased p53 transcriptional activity. Importantly, the knockdown of TIGAR by siRNA partially removed the anti-proliferative effects of NSC59984 and reduced G6PD levels in ESO26-R248W cells (**Figures 7E, F**), suggesting that p53-dependent TIGAR may be important for the metabolic effects of NSC59984 treatment and may drive the switch from high glycolysis levels to the PPP. This switch would lead cells away from glycolysis-driven proliferation and towards synthesis of nucleotides used for RNA and DNA synthesis (64) and antioxidant defense (65).

Indirect mechanisms may also be important for the observed changes in PPP function. Mutant p53 is known to form heterotetramers with p73, whereas WT p53 does not generally bind to it (66). This mutant p53 complex serves to sequester p73 and inhibit its activity (67, 68), including regulation of G6PD (57), enhancement of the PPP (69, 70), and other aspects of metabolism (71) and cell signaling (53, 72). Thus, treatment with NSC59984 may also affect mutant p53 complexation with p73, freeing p73 and thereby further increasing metabolism through the PPP. Although we did not detect a specific decrease in the amount of p73 in complex with p53-R248W upon NSC59984 treatment, the observed increase in total p73 levels are consistent with a decrease in the proportion of p73 bound to p53-R248W (**Figure 1D** and **5H**). Furthermore, prior work on NSC59984 demonstrated increased p73 activity in two mutant p53 colorectal cell lines, DLD-1 (p53 S241F) and SW480 (p53 R273H, P309S), upon treatment with NSC59984 (39), which corroborates our data on PFKL and CaN19 (**Figures S5D-E**). Finally, one aspect of the mechanism of PRIMA-1 has been shown to be dissociation of the mutant p53 complex with p73 (58). Therefore, the effects of NSC59984 on metabolism may arise both from direct p53 transcriptional regulation and from increased p73 regulation.

Structurally and functionally, the p53 R248W mutation is less intrusive than the p53 R175H mutation (73). The conformation of p53 R248W protein not only maintains many WT p53 functions but also allows the protein to continue to bind other molecules (4,^74^). We hypothesize that only small changes in p53 R248W conformation would be required to regain WT activity. p53 R248W may be more responsive to NSC59984 because it is closer to the WT form, explaining the increased effects observed in cells with the R248W mutation as compared with the R175H mutation. As the R248W mutation affects DNA binding by p53, the observed increases in p53 occupancy at the p21 and TIGAR promoters after NSC59984 treatment in ESO26-R248W cells suggest that treatment does restore some wildtype activity in this mutant.

**Figure 9:**
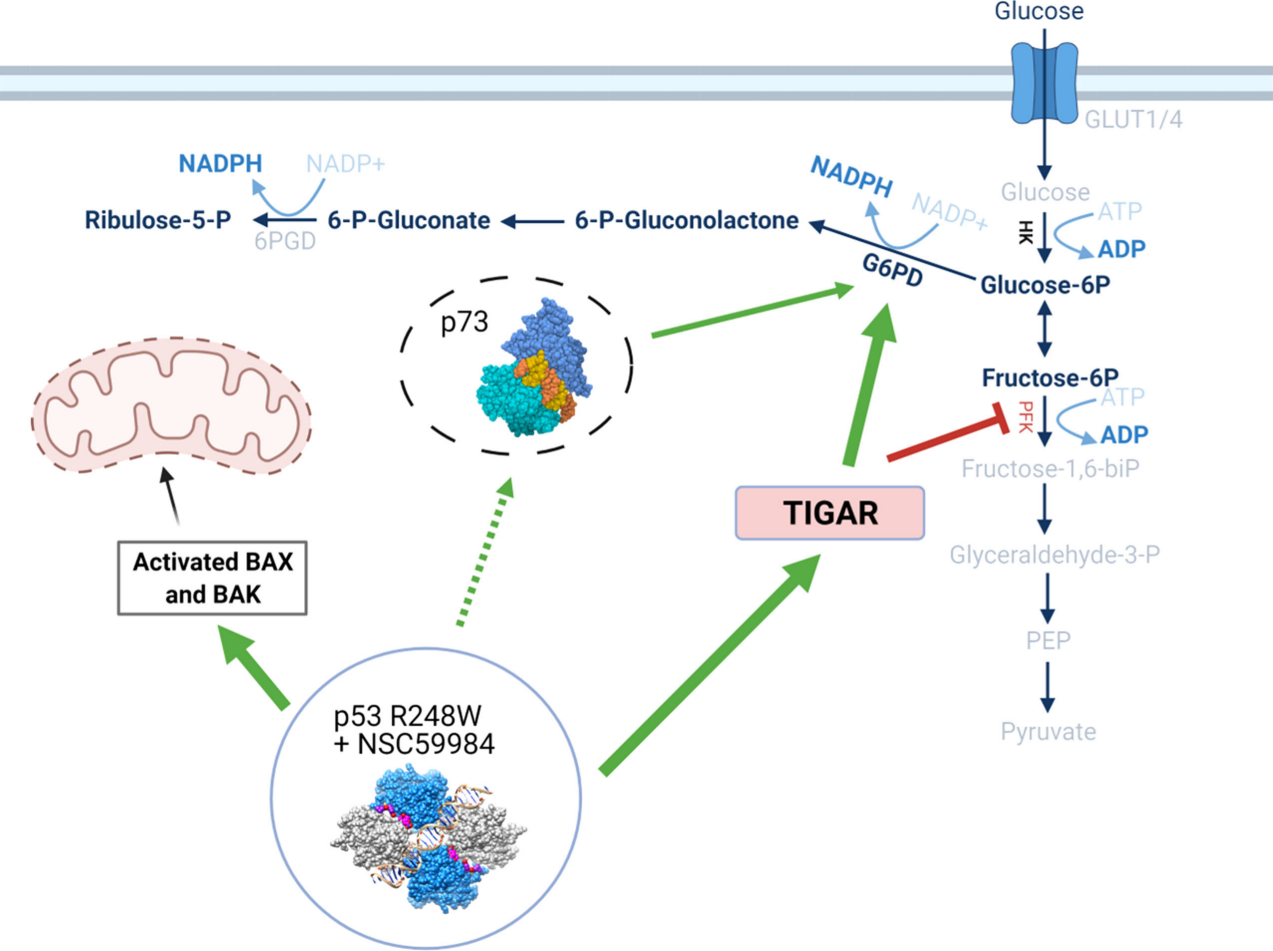
Schematic detailing the effects of p53 stabilization on metabolic pathways. The stabilization of the p53 structure upon treatment with NSC59984 initiates a cascade of effects through both transcriptional- and non-transcriptional regulation. *Via* transcriptional regulation, stabilized p53 increases expression of TIGAR which causes a subsequent reduction of PFK and blocks glycolysis. A concurrent increase in G6PD potentially mediated by p73 having been released from an inhibitory complex with mutant p53 (dashed line) allows the processing of glucose to flow through the PPP *via* increased HK and Glucose-6P. This reduces cellular energetics and subsequently cellular proliferation. Simultaneously, a transcriptional increase in Bax allows increased cytochrome *C* release from the mitochondria with ensuing intrinsic apoptosis. Labels in BOLD indicate measured increases following treatment with NSC59984. * = 0.05, ** = 0.005, *** = 0.0005, **** = 0.00005.

This work has identified a distinctive mode of action for p53 reactivation that not only results in transcriptional activity, but also has a unique effect on cellular energetics (**Figure 9**). Reactivation of p53 led to the increased expression of p53 transcriptionally-regulated genes such as p21 for cell cycle regulation, Bax for regulation of intrinsic apoptosis, and TIGAR for the regulation of glycolysis. Additionally, we show an increase in G6PD activity with a concurrent increase in HK2 and a decrease in PFK1, which in combination indicate the processing of glucose switched from glycolysis to the PPP. This switch can be attributed to the increase in TIGAR expression inhibiting the glycolysis pathway, which would lead to the reduction in proliferation due to reduced cellular energetics.

Targeting cellular metabolism is a well-established strategy for chemotherapeutics (75–78). Both PRIMA-1 and APR-246 have been shown to modulate the balance of reactive oxygen species (ROS) and reduced glutathione (GSH), allowing the accumulation of ROS and the pro-apoptotic p53 target Noxa (55, 79–82). Additionally, NSC59984 has recently been shown to increase cellular ROS levels (49). Our data supports these findings and in addition we have shown how co-stimulaton with either BSO or NAC can increase or inhibit ROS production, respectively. Thus, targeting mutant p53-containing tumors could represent a new mechanism to inhibit tumors by targeting cellular metabolism (83, 84) and use of p53 reactivators in combination with specific metabolic inhibitors could prove to be even more effective (85).

We have shown that targeting the metabolic pathways of EAC cells harboring the R248W mutation provides a therapeutic window compared to WT-p53 cells, as treatment effects were selective for mutant-p53 cells and had minimal effect on WT-p53 cells. Further, combination of NSC59984 with the metabolic inhibitors metformin or 2DG completely inhibited proliferation of mutant-p53 cells while leaving WT-p53 cells unaffected (**Figure 8**). Using these therapeutically available molecules (85–88) suggests that this combination could be used in a clinically relevant setting. Additional work in *in vivo* models is required to demonstrate clinical usefulness. Although not a model of EAC, a recently reported mouse model containing p53 R245W (R248W in human) mutation could be a good system to investigate whether various inhibitors including NSC59984 affect metabolic activity in other cancer types (89).

## Conclusion

We show that both metabolic inhibition and p53 reactivation can have varied effects in different cell lines possibly due to the p53 mutation. Targeting p53 is a monumental task. Although APR-246 is suggested to have greater specificity for the R175H mutation (90), the clinical trials in which it is being studied do not require a specific p53 mutation status. Our data, as well as that of others (91, 92), indicate that drugs targeting p53 could have much stronger effects on certain mutations due to different upstream regulators or because of differing effects on protein structure (93, 94). We have demonstrated that NSC59984 reactivates WT-like activity in EAC cells harboring mutant p53 R248W with subsequent transcription-dependent and -independent effects on tumor growth. Moreover, we showed that NSC59984 targets the dysregulated metabolism in tumors and, importantly, how the many processes regulated by p53 are not compartmentalized but rather work in concert to stem tumor growth and progression.

## Data availability statement

The original contributions presented in the study are included in the article/**Supplementary Material**. Further inquiries can be directed to the corresponding author.

## Author contributions

Conceptualization, KB, TR, and EA. Experimental Design, KB, LM, SM, DC, TF, DaS, ML, TR, and EA. Experimental Work and Data Interpretation, KB, LM, DeS, XY, SM, DC, BA, YY, AL, TR, and EA. Writing, KB, LM, and EA. All authors contributed to the article and approved the submitted version.

## Footnotes

1 Deuterated 4,4-dimethyl-4-silapentane-1-sulfonic acid
