# Supplementary material for: Targeting mutant p53-R248W reactivates WT p53 function and alters the onco-metabolic profile
Source: Front Oncol. 2023 Jan 11;12:1094210. doi: 10.3389/fonc.2022.1094210 (PMC9874945; doi:10.3389/fonc.2022.1094210)
Supplement: Supplementary file 1 [file DataSheet_1.pdf]

Brown K. et al Supplemental Tables

Table 1: p21

p21	p53						IgGm					
CP-A Carrier	31.03	31.29	31.07	31.01	31.54	31.02	35.84	36.09	35.89	36.18	37.02	36.86
CP-A NSC59984	31.0	29.48	29.64	30.62	29.47	29.36	36.37	35.67	35.89	35.41	36.14	35.69
ESO26 Carrier	35.91	35.69	35.42	31.25	30.98	31.36	54.98	55.97	56.54	58.27	59.69	58.53
ESO26 NSC59984	29.85	30.18	30.07	30.09	30.53	30.43	58.26	59.34	59.26	59.68	58.98	58.95

Table 2: TIGAR

TIGAR	p53						IgGm					
CP-A Carrier	34.30	34.10	33.48	34.03	33.63	33.67	36.69	37.86	38.93	38.00	36.10	37.68
CP-A NSC59984	34.28	32.87	31.73	33.79	33.11	32.66	36.78	37.02	36.94	36.10	37.73	36.67
ESO26 Carrier	34.265	34.673	34.125	35.483	36.878	36.131	58.521	58.520	59.723	57.669	57.115	57.702
ESO26 NSC59984	32.472	32.597	32.764	33.867	33.675	33.699	58.369	58.789	58.458	58.235	58.265	58.458

Table 3: CaN19

CaN19	p73						IgGr					
CP-A Carrier	37.12	36.73	36.14	43.05	44.53	41.99	44.27	43.02	41.53	49.28	45.37	50.03
CP-A NSC59984	42.72	39.14	37.37	37.16	37.57	37.39	46.81	52.95	41.84	47.02	44.09	54.04
ESO26 Carrier	39.811	39.868	39.341	38.516	38.740	39.670	58.538	58.487	58.470	55.645	50.196	52.875
ESO26 NSC59984	37.181	37.688	37.341	36.786	36.723	37.199	58.341	59.012	59.376	54.369	53.158	50.953

Supplemental Tables: Raw Ct values for CHiP data for p21, TIGAR and CaN19.
