# Supplementary figures and images for: Targeting mutant p53-R248W reactivates WT p53 function and alters the onco-metabolic profile
Source: Front Oncol. 2023 Jan 11;12:1094210. doi: 10.3389/fonc.2022.1094210 (PMC9874945; doi:10.3389/fonc.2022.1094210)

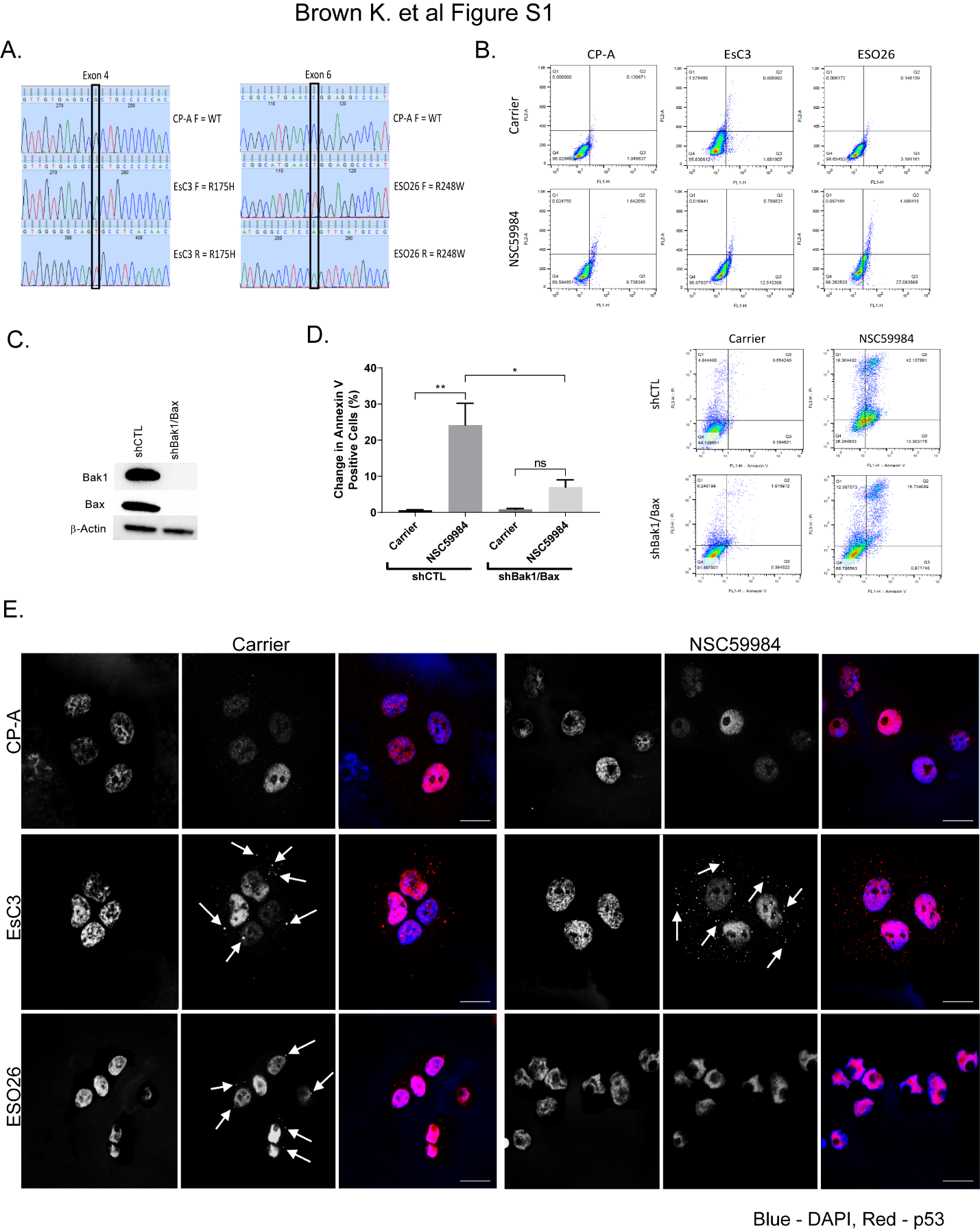


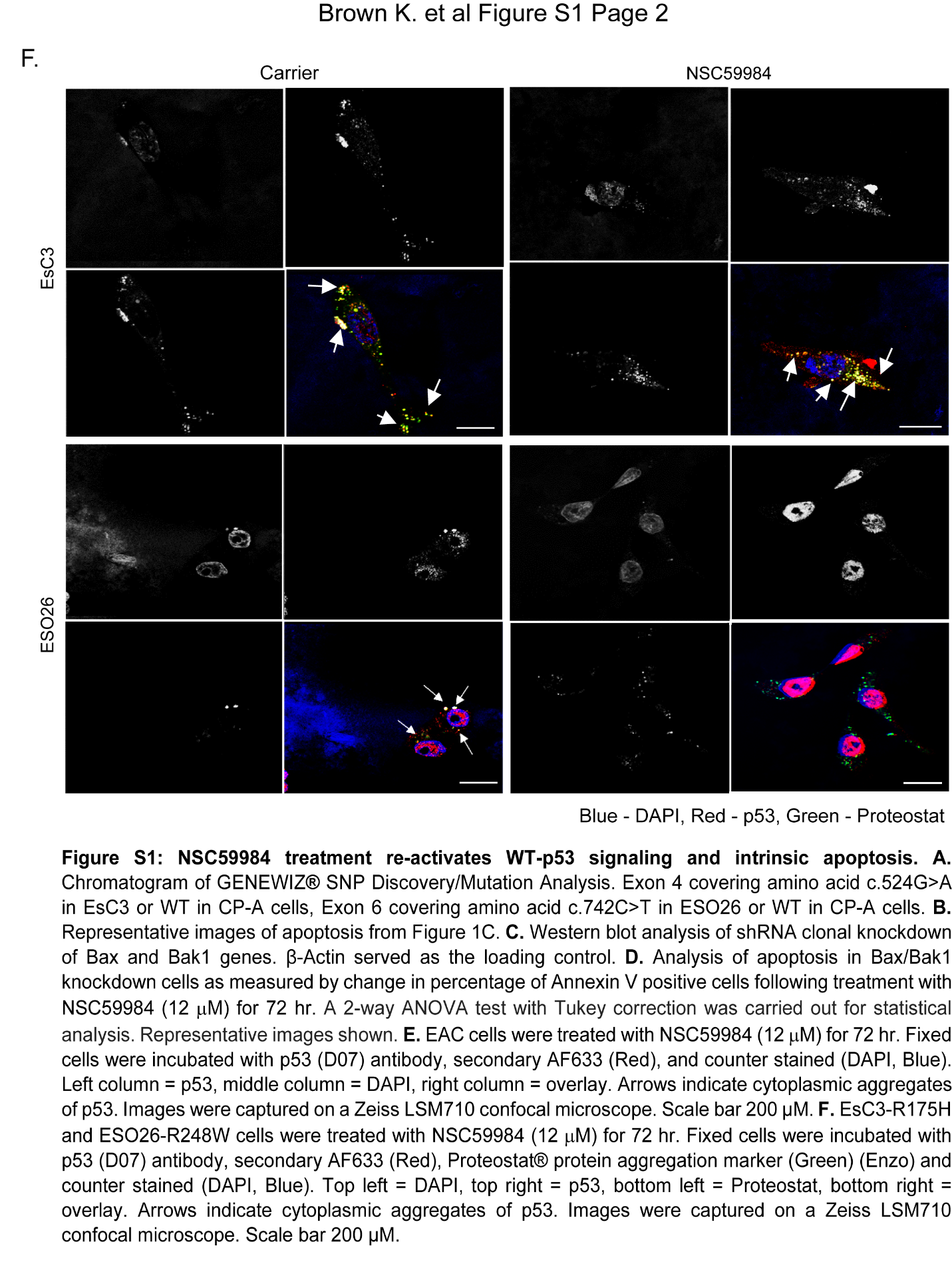


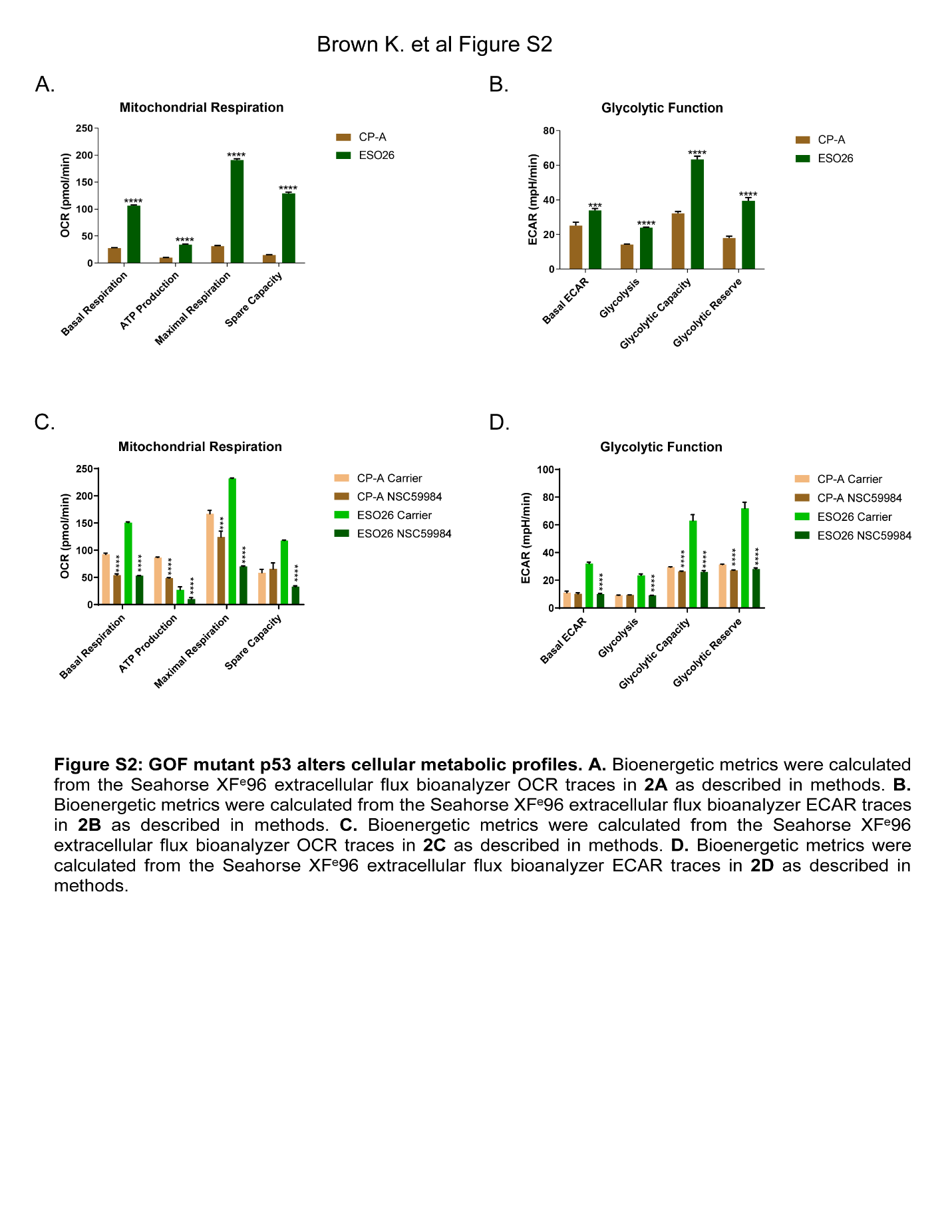


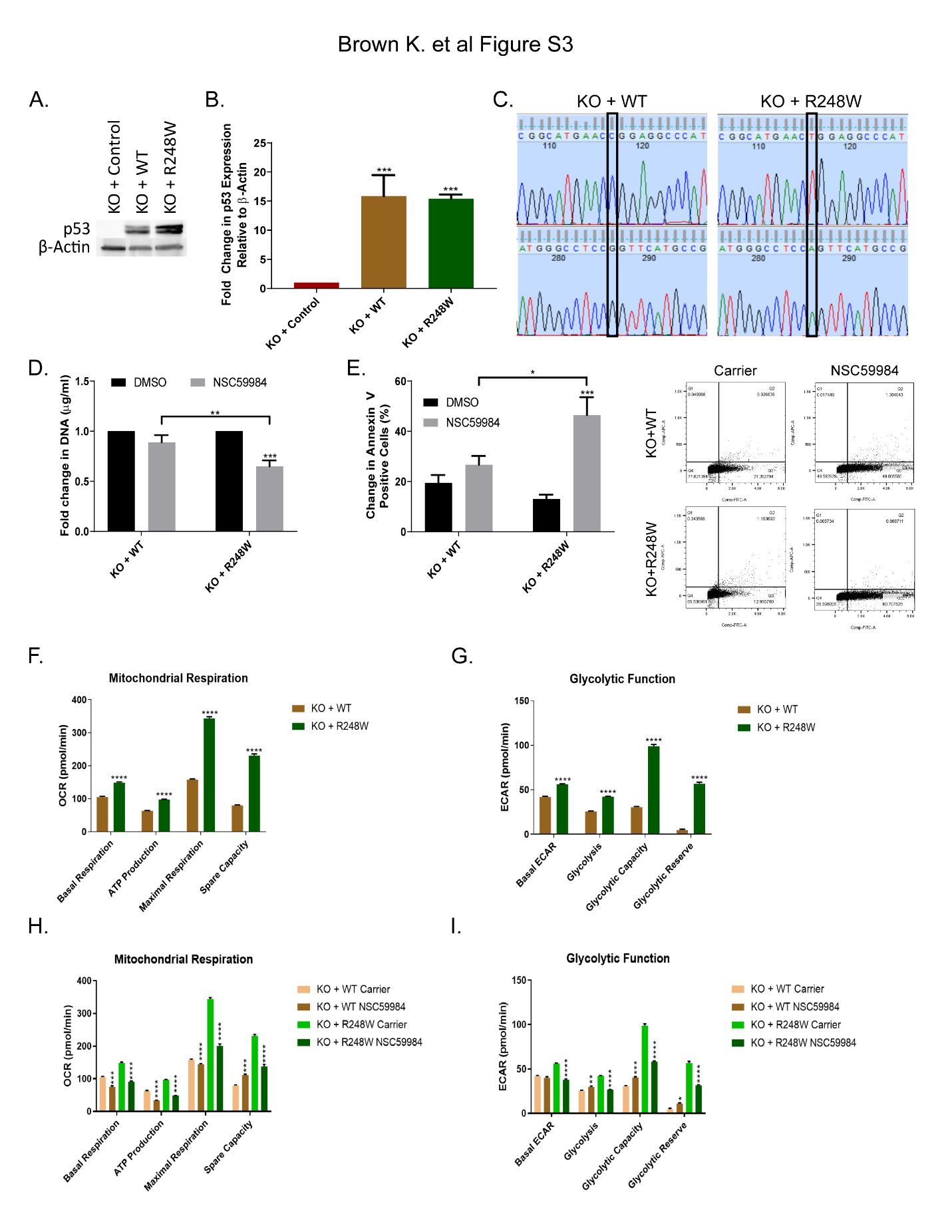


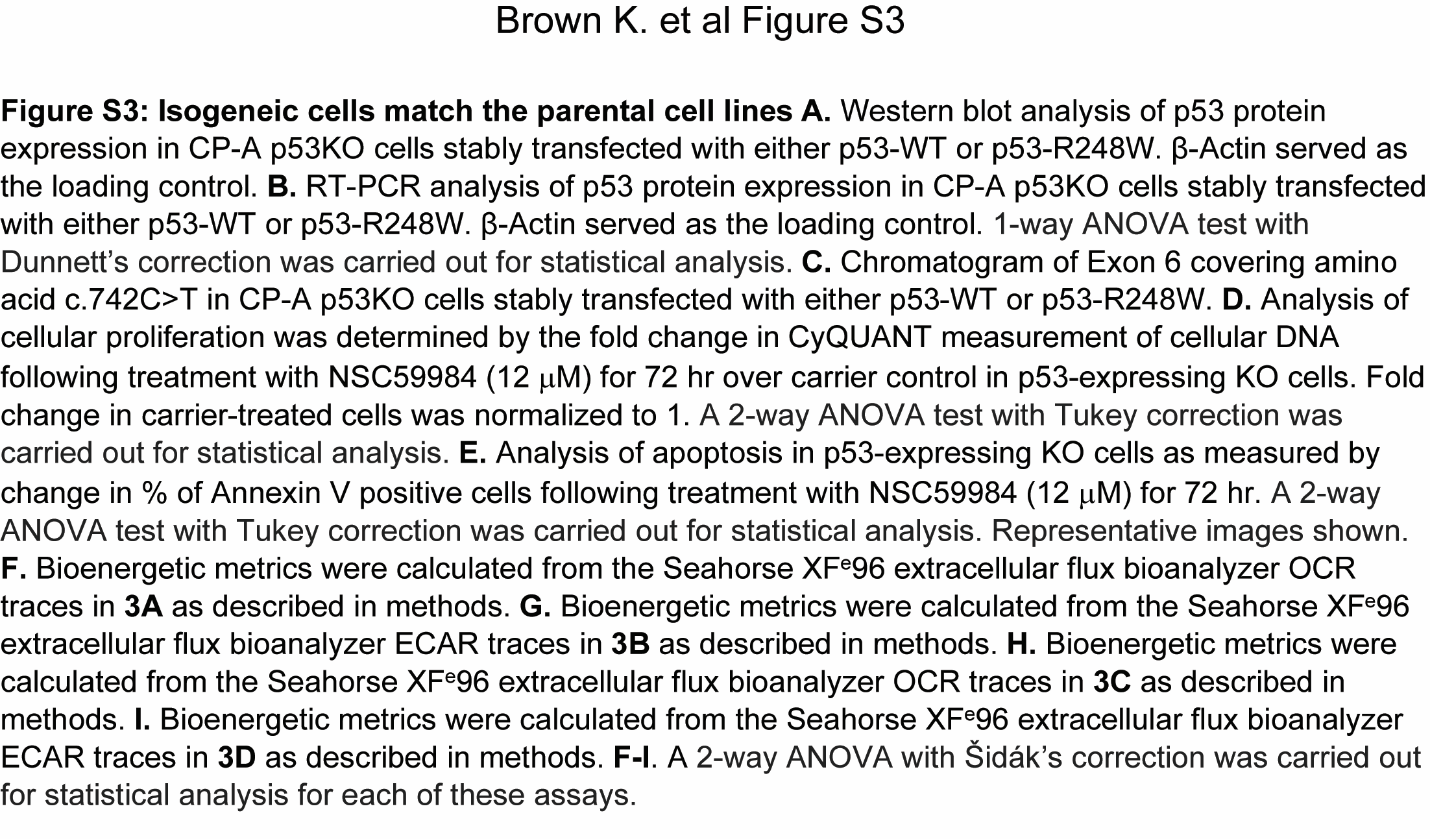


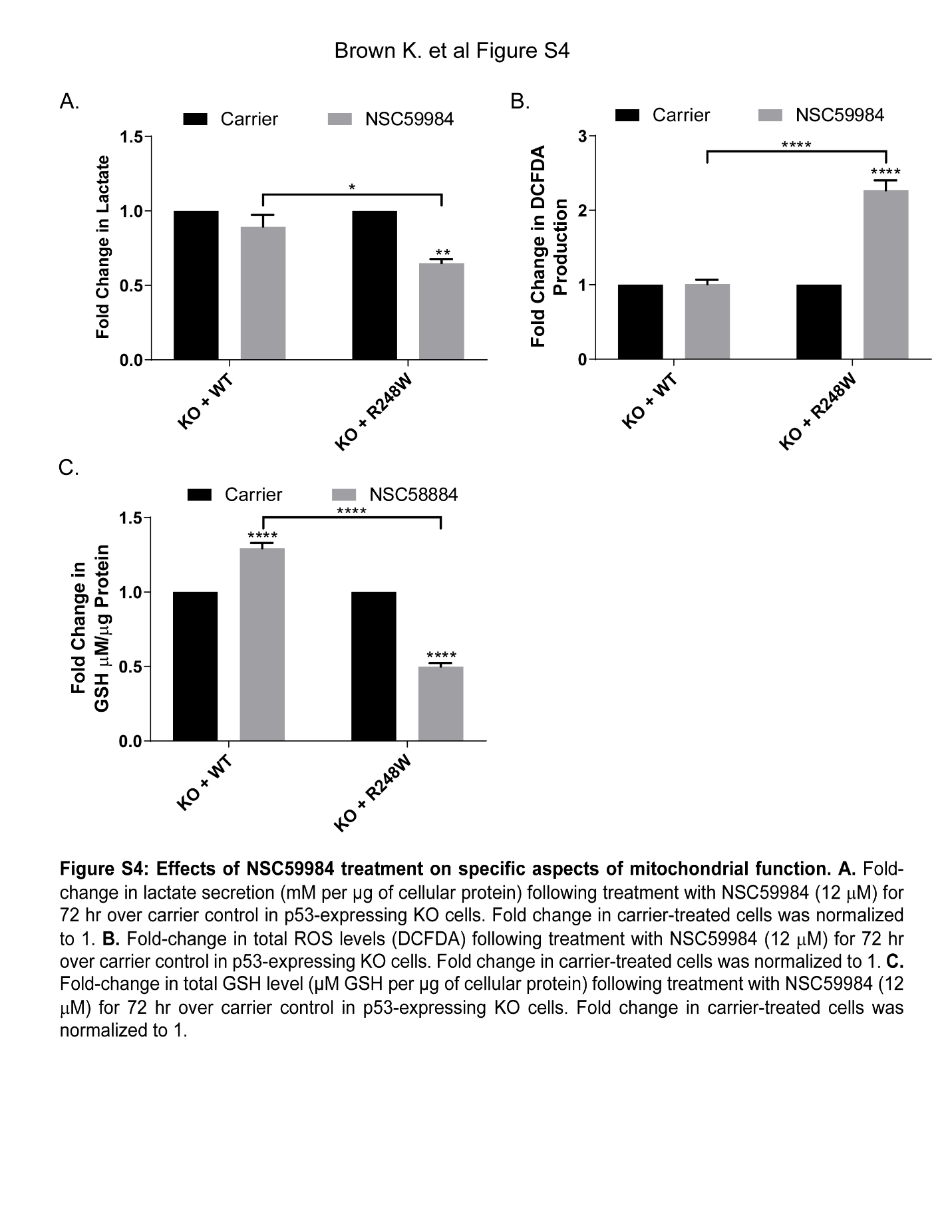


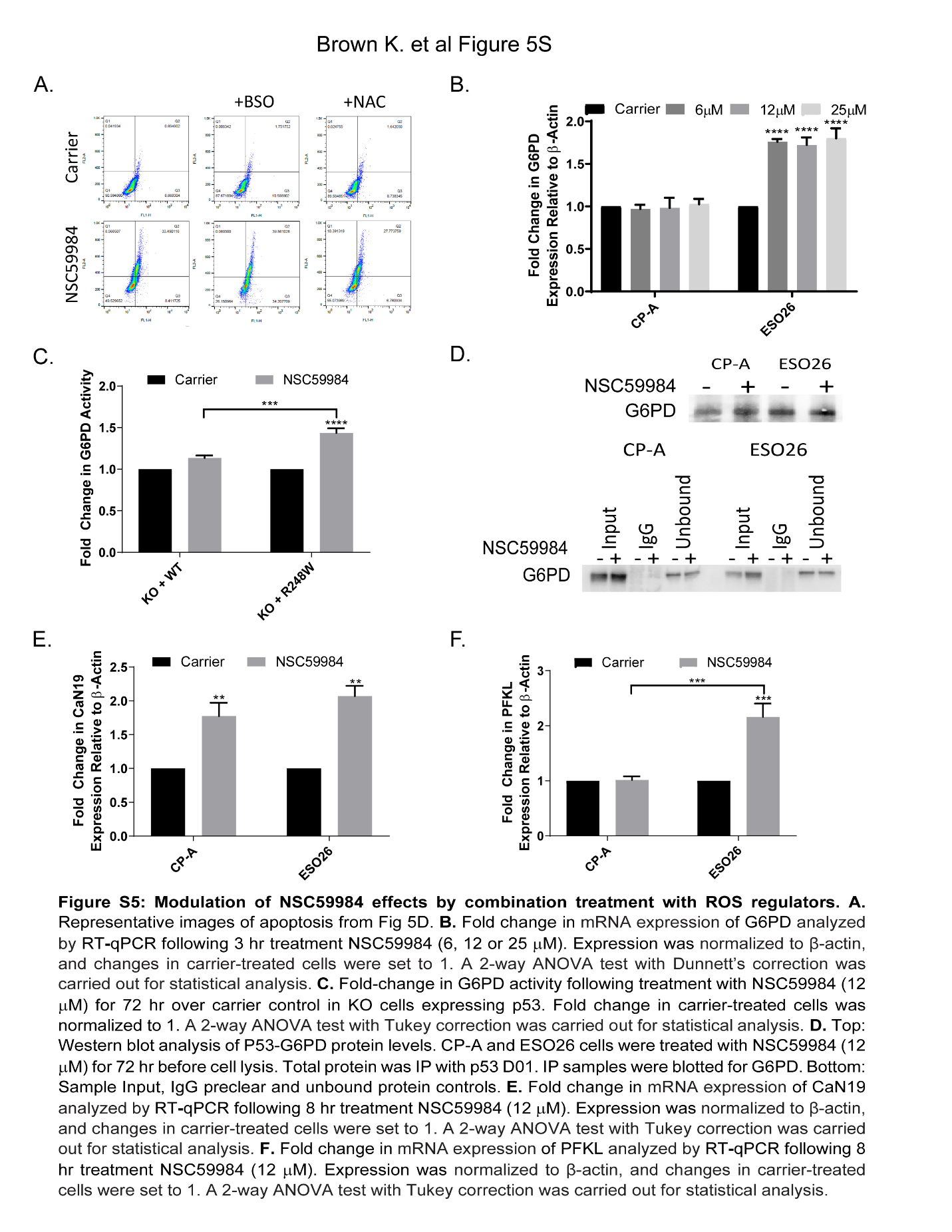


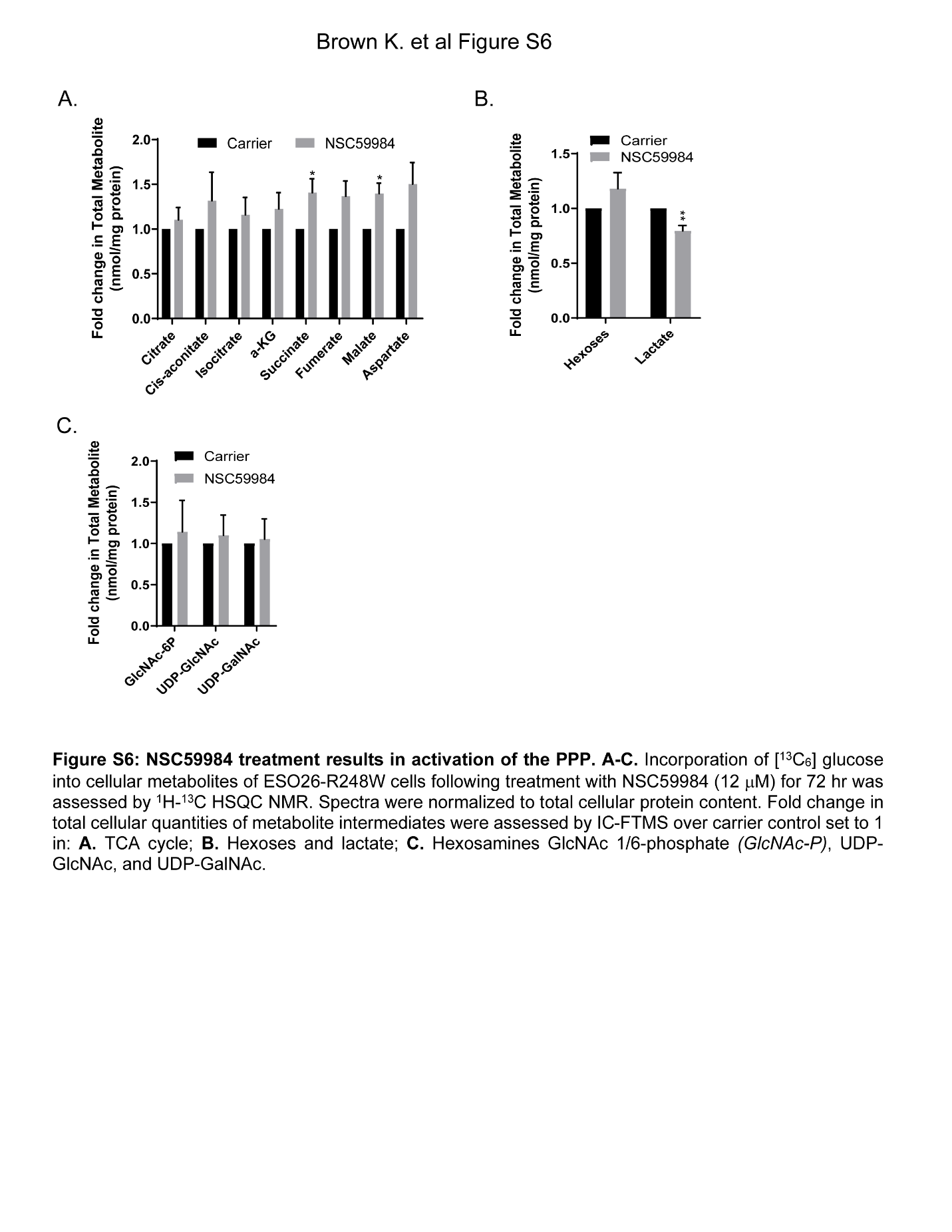


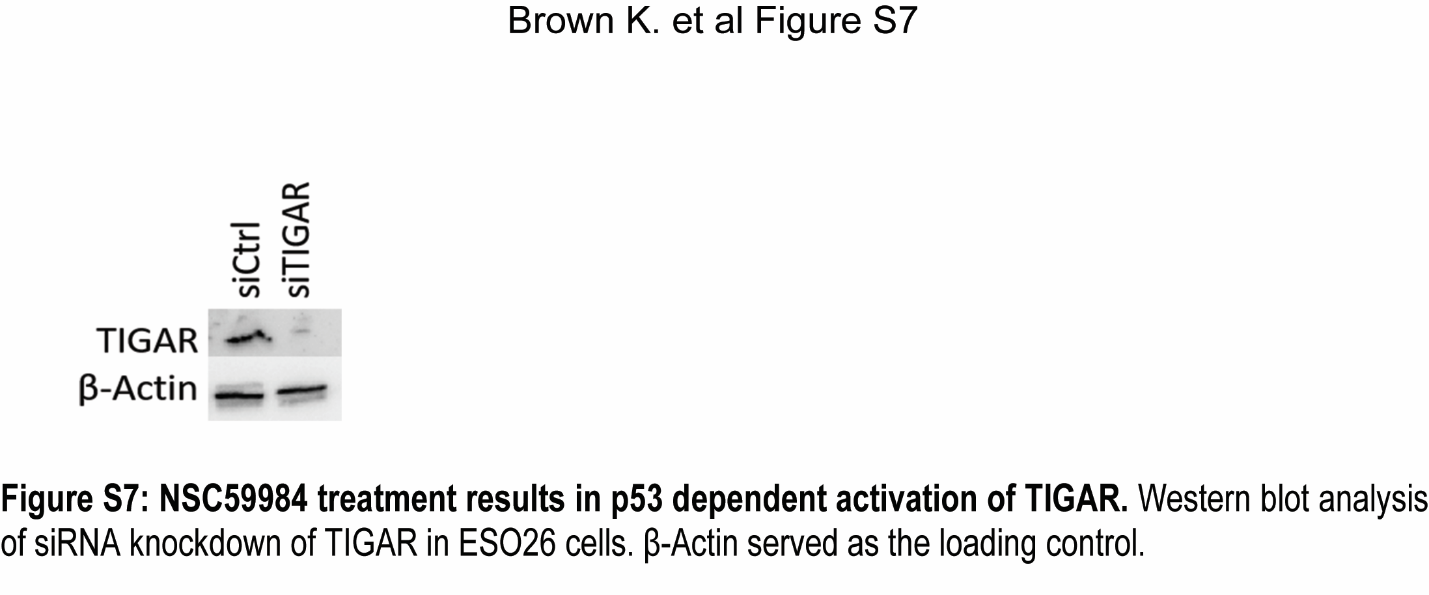

Supplement: Supplementary file 2 [file DataSheet_2.docx]
